# Differing coronavirus genres alter shared host signaling pathways upon viral infection

**DOI:** 10.1038/s41598-022-13396-7

**Published:** 2022-06-13

**Authors:** Diana Cruz-Pulido, Wilberforce Zachary Ouma, Scott P. Kenney

**Affiliations:** 1 Department of Veterinary Preventive Medicine, The Ohio State University, Columbus, OH 43210 USA; 2 Department of Animal Sciences, Center for Food Animal Health, The Ohio State University, Wooster, OH 44691 USA; 3 The Ohio Supercomputer Center (OSC), Columbus, OH 43212, USA

**Keywords:** Computational biology and bioinformatics, Cellular signalling networks, Gene ontology, Genome informatics, SARS-CoV-2, Viral host response, Virus-host interactions

## Abstract

Coronaviruses are important viral pathogens across a range of animal species including humans. They have a high potential for cross-species transmission as evidenced by the emergence of COVID-19 and may be the origin of future pandemics. There is therefore an urgent need to study coronaviruses in depth and to identify new therapeutic targets. This study shows that distant coronaviruses such as Alpha-, Beta-, and Deltacoronaviruses can share common host immune associated pathways and genes. Differentially expressed genes (DEGs) in the transcription profile of epithelial cell lines infected with swine acute diarrhea syndrome, severe acute respiratory syndrome coronavirus 2, or porcine deltacoronavirus, showed that DEGs within 10 common immune associated pathways were upregulated upon infection. Twenty Three pathways and 21 DEGs across 10 immune response associated pathways were shared by these viruses. These 21 DEGs can serve as focused targets for therapeutics against newly emerging coronaviruses. We were able to show that even though there is a positive correlation between PDCoV and SARS-CoV-2 infections, these viruses could be using different strategies for efficient replication in their cells from their natural hosts. To the best of our knowledge, this is the first report of comparative host transcriptome analysis across distant coronavirus genres.

## Introduction

Coronaviruses (CoV) are the cause of respiratory and intestinal infections in animals and humans^1^. For a relatively long period of time, coronaviruses were not considered a major human concern. This however changed with the outbreak of severe acute respiratory syndrome (SARS) in 2002 and 2003 in Guangdong, China^1,2^. SARS which emerged from the bat vector through intermediate animal hosts made it into a human transmission chain, infected at least 8096 people in 29 countries and killed 774 individuals^3,4^. A decade later, the Middle East respiratory syndrome coronavirus (MERS-CoV)—a highly pathogenic coronavirus—appeared on the Arabian peninsula^1,5^ As a result, more than 2578 people in 27 countries were infected, and at least 888 people killed by October 2021 (WHO)^3^. On December 8, 2019, the latest outbreak of a new coronavirus called sudden acute respiratory coronavirus 2 (SARS-CoV-2) was detected in Wuhan, China^6^. SARS-CoV-2 causes the coronavirus disease 2019 (COVID-19). This outbreak—the third major human coronavirus outbreak in the last two decades—has resulted in a significant societal impact and the first in the twenty-first century to reach every continent on the planet^3^. As of January 2022, over 367 million confirmed cases with more than 5.6 million deaths have occurred worldwide and continues to grow^7^.

Coronaviruses are positive-stranded RNA viruses whose genome size is about 30 kilobases^8,9^. Their genome contains genes encoding 4 structural proteins: membrane (M), nucleocapsid (N), Spike (S), and envelope (E)^3^. Coronaviruses belong to the order *Nidovirales, Coronaviridae* family, subfamily Orthocoronavirinae^1,3,10^. Four genera are found in the Orthocoronavirinae subfamily: Alphacoronavirus, Betacoronavirus, Deltacoronavirus, and Gammacoronavirus (ICTV, 2011)^1,3^. The alphacoronaviruses and betacoronaviruses are known to exclusively infect mammals, typically causing respiratory illness and gastroenteritis. Of the betacoronaviruses, there are three that cause severe respiratory disease in humans: SARS-CoV, MERS-CoV and SARS-CoV-2. The other four human coronaviruses, HCoV-NL63(alpha), HCoV-229E(alpha), HCoV-OC43(beta) and HKU1(beta), typically induce mild upper respiratory diseases in immunocompetent hosts, but can cause severe infections in elderly people, infants, and young children^1^. SARS-CoV-1, HCoV-NL63 and SARS-CoV-2 use angiotensin—converting enzyme 2 (ACE2) as a receptor and primarily infect ciliated bronchial epithelial cells^1,3^; while MERS-CoV infects unciliated bronchial epithelial cells, by employing the use of dipeptidyl peptidase 4 (DPP4) as a receptor^1,11^. SARS-CoV is thought to have been transmitted to humans from market civets, while MERS-CoV from dromedary camels^1^. Presently, the origins of SARS-CoV-2 are unclear. However, the 2002 SARS-CoV-1 outbreak—and the entry of SARS-CoV-2 into the human population—has been implicated on the wild animal handling practices common in Southern China^3^.

Gamma- and delta- coronaviruses primarily infect birds, but cases of mammalian infections have been documented^1,12–15^. CoVs can also have devastating effects in livestock populations, particularly pigs. CoVs with swine health implications include transmissible gastroenteritis virus (TGEV), porcine epidemic diarrhea virus (PEDV), porcine deltacoronavirus (PDCoV), and swine acute diarrhea syndrome virus (SADS-CoV), among others^1,16,17^. The Deltacoronavirus genus comprises mostly avian CoV pathogens of songbirds including HKU11 (bulbul coronavirus), HKU12 (thrush coronavirus), and HKU13 (munia coronavirus)^18^. PDCoV is an emerging viral disease of swine circulating globally with mortality in up to 40% of infected neonatal pigs^19^. PDCoV’s usage of an interspecies conserved amino acid domain within aminopeptidase N (APN) (also known as CD13) as a binding receptor allows infection of a diverse range of species^13,20^. Lednicky et al., identified PDCoV strains in plasma samples of three Haitian children with acute undifferentiated febrile illness^12^ confirming the ability of PDCoV to cause disease in humans.

SADS-CoV, a swine enteric alphacoronavirus, is a recent spillover from bats to pigs^1^. SADS is a highly pathogenic enteric CoV first reported in a fatal diarrhea outbreak in Guangdong province, China, in January 2017, causing the deaths of 24,693 newborn piglets^21^. This disease is caused by a novel strain of Rhinolophus bat coronavirus HKU2^1,17^. Zhou et al., provided significant evidence that the causative agent of SADS-CoV is a novel HKU2-related coronavirus that has 98.48% identity in genome sequence to HKU2^17^.

Transcriptomic analysis is a powerful application of Next Generation Sequencing (NGS) technology that allows the identification of pivotal genes and/or signaling pathways as well as biomarker and drug discovery for various diseases and novel therapeutics^22^. During the last 10 years, several studies have been focused on host cell transcriptome changes related to coronavirus infections. As an illustration of the alphacoronaviruses, Zhang et al. provided the first report of the transcriptional expression of host cells during SADS-CoV infection^21^. Hu et al. implemented a transcriptomic analysis describing the host genetic response to porcine epidemic diarrhea (PEDV) in IPEC-J2 cells^23^. Song et al. provided a transcriptomic analysis of coinfection of porcine IPEC-J2 cells with PEDV and transmissible gastroenteritis virus (TGEV)^24^. Additional transcriptome analyses have been generated in Vero E6 cells infected with PEDV^25^ and SADS-CoV^26^. Similarly, Friedman et al. used NGS for identifying the transcriptomic changes in human MRC-5 cells infected with human coronavirus (HCoV)-229E^27^. For betacoronaviruses, Blanco-Melo et al. provided a comparison of the transcriptional response of SARS-CoV-2 with other respiratory viruses to identify transcriptional factors that can determine COVID-19 biology^28^. Sun et al. established the host response patterns for SARS-CoV-2 at different time points of infection and performed a comprehensive analysis of their transcriptomic profile with SARS-CoV and MERS-CoV^29^. Yuan et.al. were able to generate the transcriptomic profile of human bronchial epithelial Calu-3 cells infected with MERS-CoV in order to investigate the importance of lipid metabolism in human viral infections^30^. Yoshikawa et al. analyzed the global genes responses of 2B4 cells infected with SARS-CoV at different time points by microarray analysis^31^. For deltacoronavirus, Cruz-Pulido et al. performed the first transcriptome analysis of human intestinal cell lines infected by PDCoV^32^. Liu et al. provided a transcriptomic profiling of long non-coding RNAs (lncRNAs) in swine testicular (ST) cells infected with PDCoV^33^. Finally, for gammacoronaviruses, Lee et al. investigated changes in chicken embryonic kidney (CEK) cells infected with infectious bronchitis virus (IBV) by transcriptome analysis^34^.

As a result of the increasing availability of transcriptomic data, it is possible to identify different transcriptomic datasets under similar disease and control conditions that can help to elucidate novel pathways and genes with remarkable accuracy^22^. Therefore, transcriptomic analyses using different datasets are becoming increasingly useful. Krishnamoorthy et al. conducted a transcriptome meta-analysis of SARS-CoV-2, SARS-CoV, and MERS-CoV at different time points of infection in order to identify potential drugs for COVID-19 treatment^22^. Coden, et al. implemented a comparative study of epithelial expression of SARS-CoV-2, SARS-CoV, MERS-CoV, HCoV-229E and Influenza in patients with asthma^35^. Alsamman et al., compared the transcriptomic data of SARS-CoV-2 to MERS-CoV, SARS-CoV, H1N1 and Ebola virus (EBOV) in order to provide valid targets for potential therapy against SARS-CoV-2^36^. Feng et al., investigated the role of hypergraph models of biological networks that are inferred from transcriptomic data (Ebola Virus, Influenza Virus, MERS-CoV, SARS-CoV and West Nile Virus) for the identification of critical genes in viral infection^37^. There are also other transcriptomic studies in blood samples of patients infected with EBOV^38^, and microarray analyses in children^39^ and patients with influenza H1N1/2009^40^.

This study compares the transcriptomic profiles of epithelial cells infected with distant CoV such as: human intestinal epithelial cells  (HIECs) infected with PDCoV^21^, normal human bronchial epithelial (NHBE) cells infected with SARS-CoV-2^41^, and porcine intestinal epithelial cells (IPEC-J2) infected with SADS-CoV^22^. We hypothesized that similar and unique aspects in the immune-associated response to coronavirus infection in epithelial cell lines exist. Comparison of the host response to highly pathogenic coronaviruses versus potential emerging human pathogen PDCoV will provide key knowledge in understanding and developing therapeutic targets for these diseases. To explore and compare the transcriptome profiles of epithelial cell lines infected by PDCoV, SARS-CoV-2, and SADS-CoV infection, this study was able to identify common differentially expressed genes (DEGs) and signaling pathways among phylogenetically distant CoV. To our knowledge, this is a novel comparative transcriptomic analysis of epithelial cells lines infected by coronaviruses differing at the genus level.

## Results

### Alpha-, beta-, and deltacoronavirus infections result in more differentially upregulated genes across 10 common immune response associated pathways

This study was able to utilize publicly available RNA-seq libraries to compare transcriptomic profiles of HIEC cells infected with PDCoV (PRJNA690955), NHBE cells infected with SARS-CoV-2 (GSE147507), and IPEC-J2 cells infected with SADS-CoV (PRJNA622652). Using the pipeline described in the Methods section, differential expression analysis of these epithelial cell lines resulted in identification of DEGs. First, 7486 DEGs (40.97%) were identified in HIEC cells infected with PDCoV, where 4011 were upregulated and 3475 were downregulated. Second, 4982 DEGs (39.75%) were identified in NHBE cells infected with SARS-CoV-2 where 2381 were upregulated and 2601 were downregulated. Third, 8686 DEGs (61.27%) were identified in IPEC-J2 cells infected with SAD-CoV where 4455 were upregulated and 4231 were downregulated (Table 1).

**Table 1:** DEGs in HIEC cells infected with PDCoV, NHBE cells infected with SARS-CoV-2 and IPEC cells infected with SADS-CoV at 24 hpi vs no infected cells.

Cell line	Up	Down	Not Sig	Total genes	Total DEs genes
HIEC	4011	3475	10,784	18,270	7486 (40.97%)*
NHBE	2381	2601	7551	12,533	4982 (39.75%)*
IPEC	4455	4231	5491	14,177	8686 (61.27%)*

Up, upregulated genes; Down, downregulated genes; Not Sig, genes detected with no significant differences. *Percentages of total genes that are differentially expressed.

**Figure 1:**
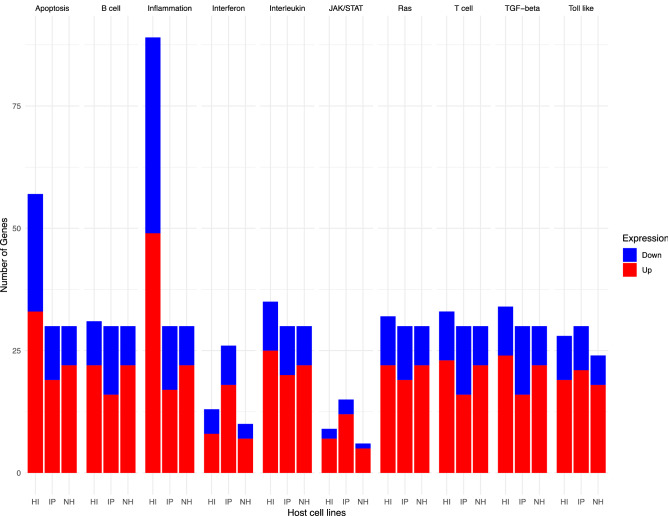
DEGs from 10 common immune-response associated pathways in HIEC (HI) cells infected with PDCoV, NHBE (NH) cells infected with SARS-CoV-2, and IPEC (IP) cells infected with SADS-CoV. Results from 10 pathways are shown: apoptosis signaling pathway, B-cell activation, inflammation/cytokine signaling pathway, interferon, interleukin signaling pathway, JAK-STAT signaling pathway, Ras signaling pathway, T-cell activation, TGF-β signaling pathway, and toll- like receptor signaling pathway. Blue is down-regulated, red is up-regulated.

We found that the DEGs are associated with 10 common immune response associated pathways. The apoptosis signaling-, interferon signaling-, interleukin signaling-, T-cell activation-, TGF-β signaling-, and Ras signaling- pathways were mostly upregulated upon viral infection. Within these pathways, more genes were affected in the inflammation/cytokine signaling pathway in all CoVs in comparison to the other 9 pathways (Fig. 1, Fig. S1–S2). In the same way, more genes were affected in HIEC cells infected with PDCoV in this pathway and a small number of genes were affected in the Interferon and Jak-Stat signaling pathway in a majority of the cell lines (Fig. 1, Fig. S8).

**Figure 2:**
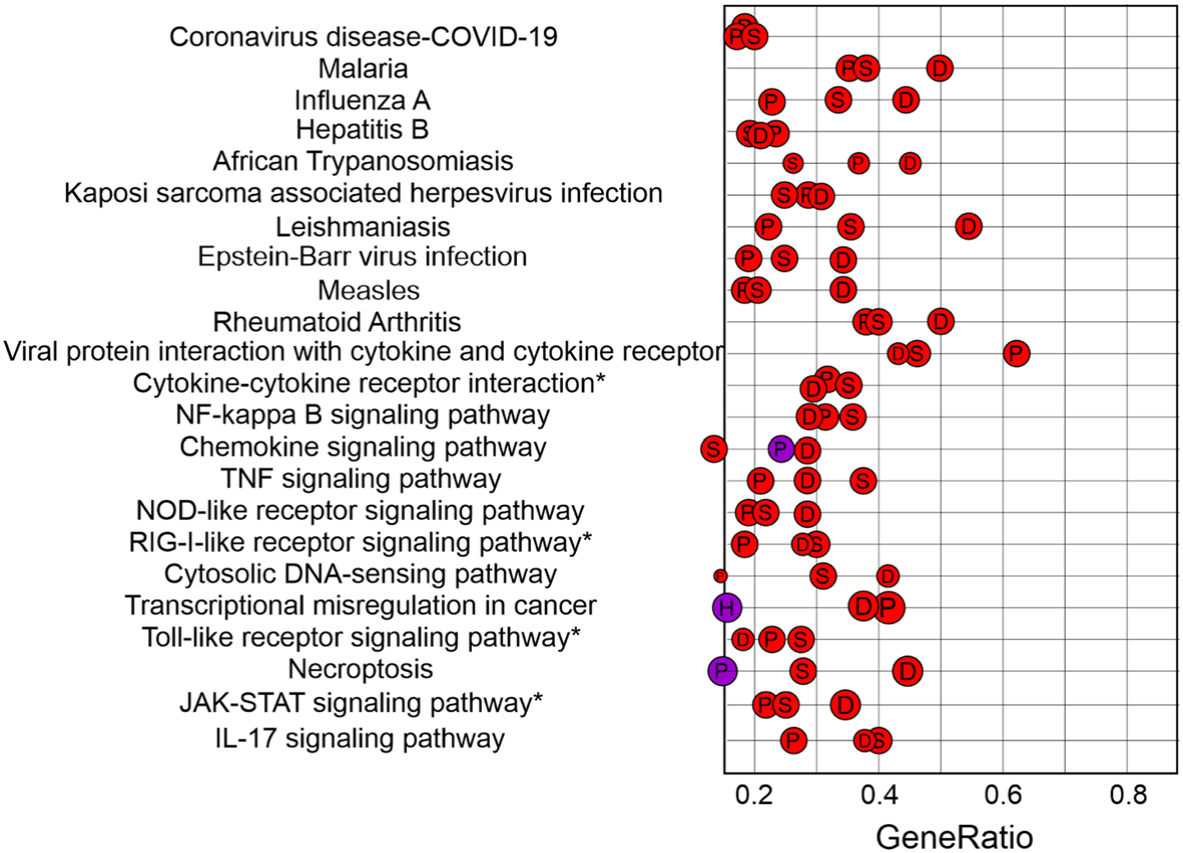
Kyoto Encyclopedia of Genes and Genomes (KEGG) gene set enrichment analysis of DEGs shared in SARS-CoV-2, PDCoV, and SADS-CoV infection in human and porcine cell lines, respectively. S = SARS-CoV-2 in NHBE cells, P = PDCoV in HIEC cells D = SADS-CoV in IPEC-J2 cells. Purple is closer to p *=* 0.05, red is closer to p = 0.01. Small circles = 20 counts, big circles = 40 counts. Asterisk = DEG pathways also in gamma CoV infection in avian cells.

DEGs of each dataset were submitted to a gene set enrichment analysis (GSEA) using Kyoto Encyclopedia of Genes and Genomes (KEGG) pathway enrichment to identify pathways that were common upon viral infection, and then, manually represented using CorelDRAW2019 (Fig. 2). A total of 23 pathways were common and enriched in human and porcine cell lines at 24 hpi (Fig. 2). These included Cytokine—Cytokine Receptor interaction pathway (Fig. S1), Viral protein interaction pathway with cytokine and cytokine receptor (Fig. S2), NF-Kappa B signaling pathway (Fig. S3), Toll like receptor signaling pathway (Fig. S4), NOD like receptor signaling pathway (Fig. S5), RIG -I like receptor signaling pathway (Fig. S6), Cytosolic DNA signaling pathway (Fig. S7), JAK-STAT signaling pathway (Fig. S8), IL-17 signaling pathway (Fig. S9), TNF signaling pathway (Fig. S10), Malaria signaling pathway (Fig. S11), Influenza A (Fig. S12), Coronavirus Disease (Fig. S13) among others (Fig. 2).

DEGs in human and pig cells also led to identification of common up-regulated and down-regulated genes between species. 826 genes were upregulated and 1,010 were downregulated among PDCoV, SARS-CoV-2, and SADS-CoV infections (Fig. S14). From these genes, 21 DEGs were common across the 10 common immune response associated pathways identified in Fig. 1 (Table 2, Fig. S15–S16). These 21 DEGs include: MAPK Activated Protein Kinase 2 (MAPKAPK2), Integrin Subunit Alpha 4 (ITGA4), Phosphatidylinositol-4,5-Bisphosphate 3-Kinase Catalytic Subunit Alpha (PIK3CA), Mitogen-Activated Protein Kinase 10 (MAPK10), Nuclear Factor Kappa B Subunit 1 (NFKB1), Cyclin Dependent Kinase Inhibitor 1A (CDKN1A), NF-Kappa-B Inhibitor Epsilon (NFKBIE), Inhibin Subunit Beta A (INHBA), TNF Receptor Associated Factor 2 (TRAF2), RELA Proto-Oncogene, NF-KB Subunit (RELA), P21 (RAC1) Activated Kinase 1 (PAK1), Baculoviral IAP Repeat Containing 3 (BIRC3), Mitogen-Activated Protein Kinase Kinase Kinase 8 (MAP3K8), Nuclear Factor Kappa B Subunit 2 (NFKB2), Interleukin 1 Receptor Associated Kinase 4 (IRAK4), Signal Transducer and Activator of Transcription 2 (STAT2), SOS Ras/Rho Guanine Nucleotide Exchange Factor 2 (SOS2), Signal Transducer and Activator of Transcription 5A (STAT5A), Suppressor of Cytokine Signaling 3 (SOCS3), Toll Like Receptor Adaptor Molecule 1 (TICAM1), and RELB Proto-Oncogene, NF-KB Subunit (RELB) (Table 2).

**Table 2:** Orthologs of upregulated and downregulated DEGs among HIEC cells infected with PDCoV, NHBE cells infected with SARS-CoV-2, and IPEC cells infected with SADS-CoV.

ID	Gene	Pathway/s	Function	References
ENSG00000162889	MAPKAPK2	Interleukin	Regulates inflammatory cytokines and apoptosis in virus-infected cells	^42,43^
ENSG00000115232	ITGA4	Inflammation	Plays an important role in inflammation	^44^
ENSG00000121879	PIK3CA	Interleukin, B cell activation, T cell activation	Shows antiviral and viral-promoting properties in infected cells	^45^
ENSG00000109339	MAPK10	Interferon	Important in integration of a number of biological processes	^46^
ENSG00000109320	NFKB1	T cell activation, Toll like receptor, B cell activation	Involved in cascades of pro-inflammatory cytokines and chemokines	^47,48^
ENSG00000124762	CDKN1A	Interleukin	Involved in cell migration and apoptosis	^49^
ENSG00000146232	NFKBIE	Toll like receptor, Inflammation	NF-κB target gene, serving as a negative feedback regulatory mechanism	^50^
ENSG00000122641	INHBA	TGF-Beta	Participates in the control of the HPG axis	^51,41^
ENSG00000127191	TRAF2	Apoptosis	Essential in homeostasis and regulation of immune cells	^52,53^
ENSG00000173039	RELA	Toll like receptor	Facilitates inflammatory and adaptive immune responses following infection	^48,54^
ENSG00000149269	PAK1	Ras, Inflammation	Major “regulator” that causes a wide variety of diseases/disorders	^55^
ENSG00000023445	BIRC3	Apoptosis	Associated to neurological diseases and apoptosis in some viral infections	^56,57^
ENSG00000107968	MAP3K8	Toll like receptor	Plays important functions in innate and adaptive immunity	^58^
ENSG00000077150	NFKB2	Toll like receptor, B cell activation	Upregulates inflammatory responses in patients with COVID-19 infection	^47,48^
ENSG00000198001	IRAK4	Toll like receptor	Leads to the production of proinflammatory cytokines	^59,60^
ENSG00000170581	STAT2	JAK/STAT	Activated by type I IFNs upon viral infection	^61^
ENSG00000100485	SOS2	T cell activation, Ras	Involved in signal transmissions that are mediated by surface protein tyrosine	^62,63^
ENSG00000126561	STAT5A	JAK/STAT	Essential for NK cell development and cytotoxicity	^45,64^
ENSG00000184557	SOCS3	Interferon	Allows optimal levels of protective immune responses against infections	^65^
ENSG00000127666	TICAM1	Toll like receptor	Involved in the anti-viral IFN response	^66^
ENSG00000104856	RELB	Apoptosis	Enhances viral transcription of some viruses in the nucleus	^67^

**Figure 3:**
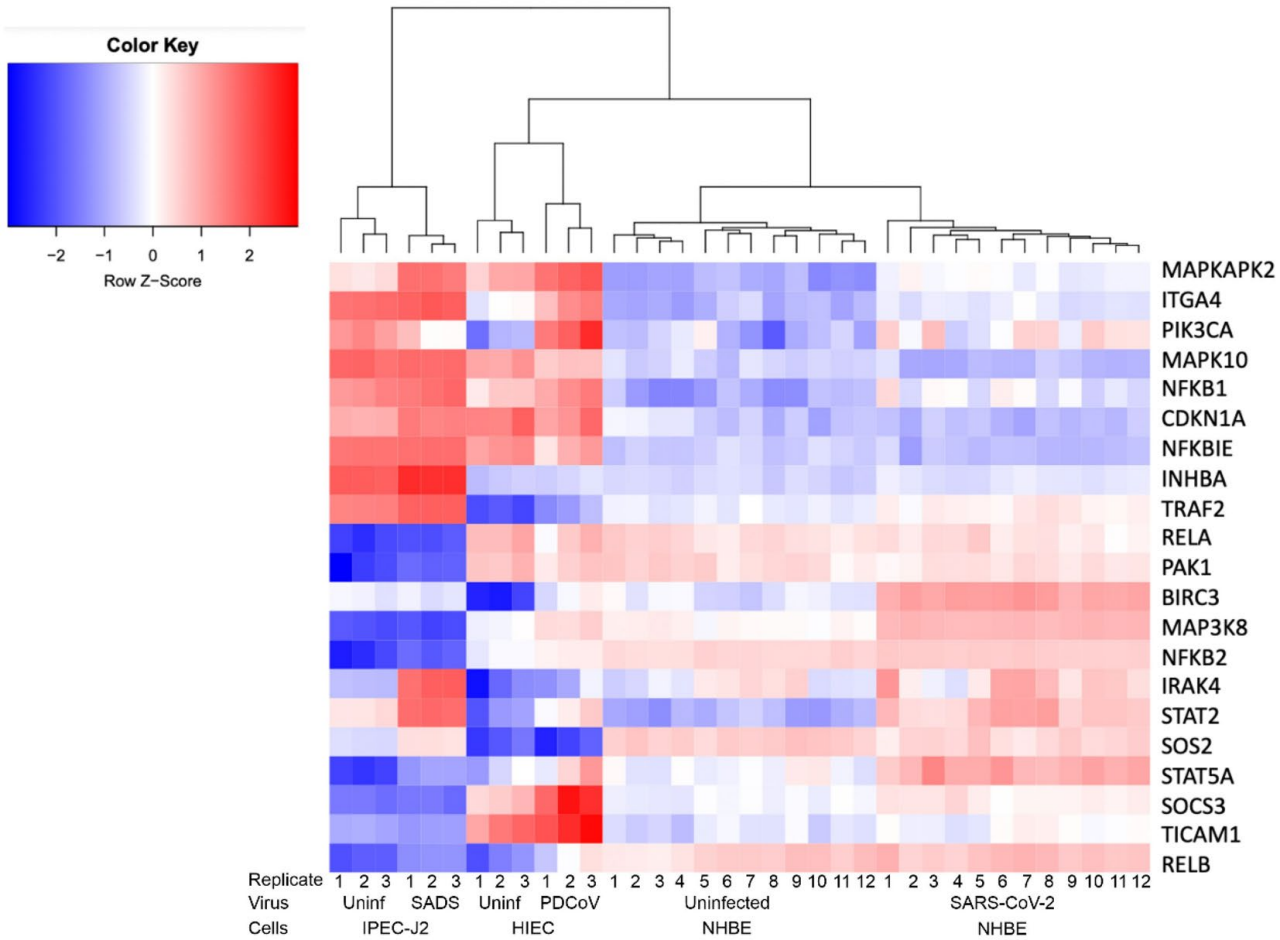
Hierarchically clustered heatmap of Orthologs of upregulated and downregulated DEGs among HIEC cells infected with PDCoV, NHBE cells infected with SARS-CoV-2 and IPEC cells infected with SADS-CoV.

Consequently, we utilized a hierarchically clustered heatmap to visualize expression profiles of these 21 DEGs (Fig. 3). We found MAPKAPK2, ITGA4, PIK3CA, MAPK10, NFKB1, CDKN1A, NFKBIE, INHBA and TRAF2 were up-regulated upon PDCoV and SADS-CoV infections but down-regulated upon SARS-CoV-2 infection. We also noted that RELA, PAK1, BIRC3, MAP3K8, NFKB2, IRAK4, STAT2, SOS2, STAT5A, SOCS3, TICAM1 and RELB were downregulated upon PDCoV and SADS-CoV infections but up-regulated upon SARS-CoV infection (Fig. 3).

### There is a positive correlation between PDCoV and SARS-CoV-2 infections

**Figure 4:**
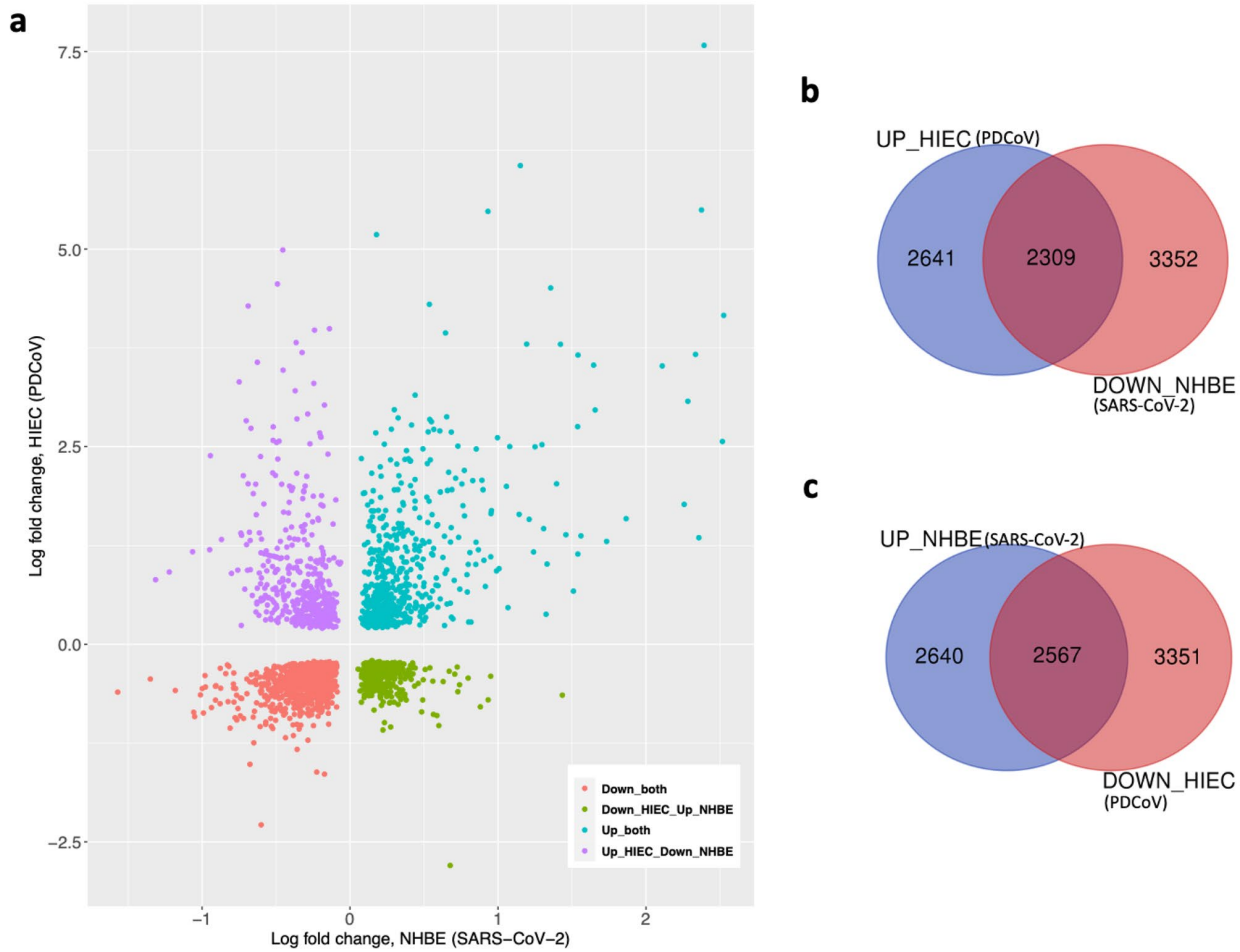
(**a**) Scatter plot of differentially up-regulated and down-regulated genes in HIEC cells infected with PDCoV and NHBE cells infected with SARS-CoV-2*. (**b**) Venn diagram of differentially up-regulated genes in HIEC cells and down-regulated genes in NHBE cells. (**c**) Venn diagram of differentially up-regulated genes in NHBE cells and down-regulated genes in HIEC cells. *Significant DEGs are represented in both datasets, in different quadrants on this graph as follows: purple dots correspond to up-regulated genes in HIEC and down-regulated genes in NHBE; blue dots show up-regulated genes in both datasets; red dots correspond to down-regulated genes in both datasets; and green dots show down-regulated genes in HIEC and up-regulated genes in NHBE.

To see if there is a relationship between PDCoV and SARS-CoV-2 infections, we first identified genes that are upregulated and/or downregulated in both datasets (Fig. 4a). We focused on the analysis of DEGs that are upregulated and downregulated in HIEC and/or NHBE datasets. As a result, we were able to identify 2309 DEGs that were upregulated in HIEC cells infected with PDCoV and down-regulated in SARS-CoV-2 infected NHBE cells (Fig. 4b). Similarly, we were able to identify 2567 DEGs that were upregulated in NHBE cells infected with SARS-CoV-2 and downregulated in HIEC cells infected with PDCoV (Fig. 4c). This study also enabled identification of common DEGs that are upregulated in HIEC cells infected with PDCoV and downregulated in NHBE cells infected with SARS-CoV-2 at 24 hpi across 9 pathways (Fig. S17). These genes include TNF Superfamily Member 10 (TNFSF10), Inositol 1,4,5-Trisphosphate Receptor Type 1 (ITPR1), Vav Guanine Nucleotide Exchange Factor 3 (VAV3), Complement C5a Receptor 1 (C5AR1), Interleukin-15 (L15), Cyclin Dependent Kinase Inhibitor 1B (CDKN1B), Forkhead Box O3 (FOXO3), Interleukin 6 Receptor (IL6R), Mitogen-activated protein kinase 6 (MAPK6), Mitogen-Activated Protein Kinase Kinase Kinase 1 (MAP3K1), Mitogen-Activated Protein Kinase 4 (MAP3K4), Phosphoinositide-3-Kinase Regulatory Subunit 1 (PIK3R1), Vav Guanine Nucleotide Exchange Factor 3 (VAV3), Cell Division Cycle 42 (CDC42), ALG5 Dolichyl-Phosphate Beta-Glucosyltransferase (ALG5), Activin A Receptor Type 2B (ACVR2B), Activin A Receptor Type 1(ACVR1) and CREB Binding Protein (CREBBP). (Table S1c, Fig. S17). Likewise, we were able to identify common DEGs that are upregulated in NHBE cells infected with PDCoV and downregulated in HIEC cells infected with SARS-CoV-2 at 24 hpi across 10 pathways (Fig. S18). These genes are Mitogen-Activated Protein Kinase 3 (MAPK3), Transmembrane BAX Inhibitor Motif Containing 6 (TMBIM6), Calmodulin 3 (CALM3), Rac Family Small GTPase 1 (RAC1), Actin Beta (ACTB), Collagen Type XII Alpha 1 Chain (COL12A), Interferon Gamma Receptor 2 (IFNGR2), Ribosomal Protein S6 Kinase A6 (RPS6KA6), Ras Homolog Family Member A (RHOA), P21 (RAC1) Activated Kinase 1 (PAK1), Transforming Growth Factor Beta Receptor 2 (TGFBR2). (Table S1d, Fig. S18). Next, we performed the correlation between the two datasets using Pearson’s correlation coefficient and we found that there is a positive correlation between PDCoV and SARS-CoV-2 infections (Fig. S19).

## Discussion

Novel coronaviruses crossing between animals and humans are likely to continue to be the source of future pandemics^68^. To anticipate therapeutics for future coronavirus outbreaks, there is an urgent need to understand potentially conserved therapeutic targets. This study was able to explore and compare the transcriptome profiles of epithelial cell lines infected by PDCoV, SARS-CoV-2, and SADS-CoV infection at 24 hpi; and to show that distant coronaviruses such as Alpha-, Beta-, and Deltacoronaviruses share common immune associated pathways and genes that could be used as conserved targets for drug discovery. Our analyses appear to be the first report of comparative transcriptome analysis of coronaviruses at the genus level, incorporating available public datasets from alpha-, beta-, and deltacoronavirus host responses. It is important to point out that the main limitation of this research was lack of public transcriptome datasets available related to cell lines infected with different coronavirus groups. Future studies need to make their transcriptomic data readily available in order to make more accurate and complete comparisons.

Through a gene level approach, we identified common DEGs in the transcription profile of each epithelial cell line infected with PDCoV, SARS-CoV-2, or SADS-CoV. These DEGs associated with 10 common immune related pathways that were mostly upregulated upon infection (Table 1, Fig. 1). These results are in line with publications reporting more than 80% of immune related genes being up-regulated^21,28,32^. Likewise, PDCoV, SARS-CoV-2, and SADS-CoV shared 21 DEGs across 10 common immune response associated pathways (Table 2, Fig. 1, Fig. S15–S16). These genes are described per group/function as follow: MAPKAPK2, also known as MK2; MAPK10 and MAP3K8 are in the first group that corresponds to genes that are part of MAPK signaling and are involved in inflammatory processes, innate and adaptive immunity^42,43,46,58^. In this group, MAPK10 has recently been shown to be downregulated in patients with Down syndrome and COVID-19^46^. Other genes that are involved in inflammatory processes include ITGA4 (also known as CD49d), RELA, IRAK4, SOCS3, and genes that belong to the NF-κB signal transduction pathway such as NFKB1and NFKBIE^44,47,48,51,54,59,60,65^. In this case, NFKB1 is associated with the upregulation of inflammatory responses in patients with COVID-19 infection^47,48^.

PIK3CA, TRAF2, RELA, STAT2 and TICAM1 are part of a second group of genes that are involved in the activation of type I IFN expression and exhibit antiviral defense properties^45,48,52–54,61,66^. In this group, RELA is involved in upregulation of inflammatory responses in patients with COVID-19 infection^48,54^. CDKN1A and BIRC3 are associated with another group of genes that are involved in apoptosis in some viral infections^49,57^.

Finally, some unique genes play key functions. For instance, PAK1 upregulation is associated with several cancers, malaria, influenza, HIV, and COVID-19^55^; and RELB enhances viral transcription of some viruses in the nucleus^67^ (Table 2). Taken together, these results suggest that many of these genes are involved in inflammatory processes, activation of type I IFN expression, apoptosis; and several of them are involved in upregulation of inflammatory responses in patients with COVID-19 infection. We also speculate that, based on visualization of expression profiles of these 21 DEGs in a heatmap (Fig. 3), PDCoV and SADS-CoV are using similar strategies for efficient viral replication in the host cells despite species differences in comparison to SARS-CoV-2. This is expected since PDCoV and SADS-CoV are both swine-adapted enteric coronaviruses.

Consequently, a pathway level analysis identified pattern recognition receptor (PRR) signaling pathways—such as Toll- like receptors (TLR) (Fig. S4), NOD like receptors (NLR) (Fig. S5), and RIG-I-like receptors (RLRs) (Fig. S6)—upon coronavirus infections. Additionally, this study has found that PDCoV, SARS-CoV-2, and SADS-CoV shared 23 pathways that included Cytokine—Cytokine receptor interaction (Fig. S1), viral protein interaction with cytokine and cytokine receptors (Fig. S2), NF-Kappa B (Fig. S3), Cytosolic DNA signaling (Fig. S7), JAK-STAT (Fig. S8), IL-17 signaling (Fig. S9), TNF signaling (Fig. S10), Malaria signaling (Fig. S11), Influenza A (Fig. S12), Coronavirus Disease (Fig. S13), among others (Fig. 2). Some of these pathways, including the JAK-STAT signaling pathway and the cytosolic DNA sensing pathway, play a pivotal role in innate immune responses^69,70^. Pathways such as Cytokine-cytokine receptor interaction and Toll-like receptor signaling have top ranked functions correlated to gamma CoV infection in avian cells (Fig. 2)^34^.

Moreover, pathways such as NF-Kappa B signaling, RIG-I like receptor signaling, NOD like receptor signaling, Cytosolic DNA sensing, Influenza A, Malaria, TNF-signaling, Cytokine-cytokine receptor interaction and Toll like receptor signaling were significantly enriched during the SADS-CoV infection^21^. GO terms associated with response to virus, response to cytokine, regulation of MAPK, programmed cell death and inflammatory response were enriched during SARS-CoV-2 infection^28^. Also, cytokine response GO terms, IL-17 signaling pathway, TNF signaling pathway, and NF-Kappa B signaling were the chief pathways associated with SARS-CoV-2^36^. In fact, NF-Kappa B has been shown to be involved in inflammatory responses upon infection with human coronaviruses. Similarly, acute respiratory failure in influenza and COVID-19 patients is associated with the upregulation of IL-6 (an interleukin that is part of Cytokine—Cytokine Receptor Interaction Pathway)^71,72^. In this study, IL-6 was also upregulated in HIEC cells infected with PDCoV and NHBE cells infected with SARS-CoV-2 (Fig. S1). Another study that compared SARS-CoV-2 with Ebola virus, H1N1 influenza virus, MERS-CoV and SARS-CoV found that genetic pathways associated with hepatitis B, the AGE-RAGE signaling system, malaria, influenza A and rheumatoid arthritis were the most significant pathways altered upon infection^36^. In our study, we also found enriched pathways related to malaria (Fig. 2, Fig. S11), hepatitis B (Fig. 2), and influenza A (Fig. 2, Fig. S12). These results indicate that pathways associated with innate immune responses are shared among different coronavirus genres upon viral infection.

The differences between DEGs that are upregulated and downregulated in HIEC and/or NHBE datasets with PDCoV and SARS-CoV-2 (Fig. 4) allow us to hypothesize that PDCoV must overcome different host-innate immune evasion strategies in human cells to be more successful in the progression of infection while SARS-CoV-2 has likely already overcome many of these barriers. We can also see this trend in the change of gene expression in different pathways that were enriched upon viral infection (Fig. S1–S13). These DEGs were mainly related to apoptosis signal and immune response regulation. Interestingly, furin was upregulated in NHBE cells infected with SARS-CoV-2 and down-regulated in HIEC cells infected with PDCoV. Furin has a noted role in viral pathogenesis; for coronaviruses, furin cleavage sites have been found widely in betacoronaviruses as well as in avian-origin gammacoronaviruses and certain canine and feline alphacoronaviruses (Table S1f.)^73^. MAPK3 was upregulated in NHBE cells infected with PDCoV and downregulated in HIEC cells infected with SARS-CoV-2 in almost all 10 pathways (Fig. S18 and Table S1d). Buggele et al. showed that MAPK3 and IRAK1 protein levels are reduced during influenza virus infection; while this gene, also known as ERK1^74^, can positively regulate RNA/protein synthesis in astroviruses, viral protein synthesis in alphaviruses, CVB3 replication, hepatitis C virus (HCV) genome synthesis, and avian leukosis virus replication and virus- induced tumorogenesis^75^. RAC1 was upregulated in NHBE cells infected with PDCoV and downregulated in HIEC cells infected with SARS-CoV-2 in B-cell, Inflammation, Ras and T- cell signaling pathway. RAC1 plays an important role in the regulation of virus entry, replication, and release^76–79^ and the inhibition of this gene leads to enhanced virus production^76,80^. CALM3 that was also upregulated in NHBE cells infected with PDCoV and downregulated in HIEC cells infected with SARS-CoV-2, was found in B and T cell activation pathways. A study has found that inhibition of Calmodulin-Dependent Kinase Kinase blocks human cytomegalovirus and severely attenuates production of viral progeny^81^. Nevertheless, the response to PDCoV showed a positive correlation (Pearson correlation coefficient = 0.4625) to SARS-CoV-2 infection (Fig. S19). Positive correlation also indicates that DEGs in both datasets showed similar changes in expression and can also explain the large overlap of DEGs between these two viruses. Similar trends were found in the comparison between SARS-CoV-2 and respiratory syncytial virus (RSV)^82^. These results suggest that even though there is a positive correlation in the immune-associated response to PDCoV and SARS-CoV-2, these viruses could be using different strategies to improve viral fitness in the infected host. Further study and validation of this correlation is warranted.

In summary, host cells infected with members of the phylogenetically diverse Alpha-, Beta-, and Deltacoronavirus genres share common immune associated pathways and genes. Therapeutics modulating these pathways may be effective in treating current and future novel coronavirus outbreaks. Ten common immune associated pathways were mostly upregulated upon infection across phylogenetically divergent coronaviruses. These viruses shared 23 pathways and 21 DEGs. The 21 DEGs provide conserved targets for drug discovery and potential therapy against emerging coronaviruses. Finally, despite a positive correlation between PDCoV and SARS-CoV-2 infections, these viruses could be using different immune escape strategies. We speculate that PDCoV is currently less successful than SARS-CoV-2 at causing significant disease in humans due to a lack of human adaptation. However, there is always a possibility that PDCoV can evolve to adapt similar mechanisms that have been used by SARS-CoV-2. Incorporation of a deltacoronavirus into comparative transcriptome analysis provides an initial report of host responses during infection with coronaviruses at the genetically distant genus level.

## Methods

### Datasets

Raw sequence reads of NHBE cells infected with SARS-CoV-2, HIEC cells infected with PDCoV and IPEC cells infected with SADS-CoV were obtained from NCBI Gene Expression Omnibus (GEO) server under the accession number GSE147507^28^, Sequence Read Archive (SRA) with accession numbers PRJNA690955^32^ and PRJNA622652^21^, respectively.

### Data pre-processing and alignment

Raw sequence reads were quality checked including removal of adapter sequences by using FastQC^83^ and BBMap^84^. Reads were aligned to *Homo sapiens* GRCh38 genome release 97 (ftp://ftp.ensembl.org/pub/release-97/fasta/homo_sapiens/dna/) and *Sus scrofa* 11.1 genome release 97 (ftp://ftp.ensembl.org/pub/release-97/fasta/sus_scrofa/dna/) using the Rsubread aligner^85^. Computer code is available in GitHub (https://github.com/Diana-Ouma/Comparative-transcriptome-analysis).

### Expression data pre-processing

Gene expression counts were identified from the alignment files in BAM format using Rsubread^85^. Raw count data was transformed to counts per million (CPM) and log-CPM using EdgeR^85^. Genes that were not expressed in any biologically significant levels (CPM less than 1) were discarded. Expression values were normalized using the trimmed mean of M-values (TMM) normalization^86^.

### Differential expression

Differential expression analysis was performed between infected and mock samples in each cell line. Briefly, dispersion of each gene was first estimated, followed by fitting generalized linear models (GLM) on the expression dataset. Within the R programming environment, a quasi-likelihood (QL) F-test method was used to test for differential expression^85^ along with Limma and EdgeR statistical packages being utilized for analyses. Statistically significant DEGs between uninfected control and the infected cell lines were identified using a Bonferroni-Hochberg adjusted *P* value cut-off of 0.05 and log fold change of 1 (FDR <  = 0.1).

### GO function and KEGG pathway enrichment analysis

Function classification and enrichment analysis of DEGs were performed using KEGG Gene Set Enrichment Analysis available in the R packages ClusterProfiler^87^, and GAGE^88^ as well as using DAVID^89^ and PANTHER 15.0 software^90^. Pathway enrichment was analyzed based on the KEGG database^91^. KEGG pathways with *p* values < 0.05 were considered to be significantly enriched. Dot plots were made by the R package, ClusterProfiler^87^. KEGG pathways with DE genes were visualized and generated by the R package, Pathview^92^. KEGG pathway results produced by individual analyses were manually compared with conserved results combined into a single chart utilizing CorelDRAW2019. Intersections of common upregulated and downregulated DEGs across pathways were represented by Venn Diagrams using the draw custom Venn Diagram tool^93^.

### Correlation analysis

Correlation of expression of DEGs in mock and infected samples was performed for two datasets: HIEC cells infected with PDCoV and NHBE cells infected with SARS CoV-2. Briefly, correlation of absolute Log fold change of DEGs between the two datasets was performed with the use of the Pearson’s correlation coefficient. Additionally, scatterplots depicting correlation of Log fold changes were generated. Both the correlation analysis and generation of scatterplots were performed using the R Statistical and Programming environment.

## Supplementary Information

Supplementary Information 1.

Supplementary Information 2.

## Data Availability

The datasets analyzed during this study are available in NCBI Gene Expression Omnibus (GEO), accession number GSE147507, [https://www.ncbi.nlm.nih.gov/geo/query/acc.cgi?acc=GSE147507]; Sequence Read Archive (SRA) with accession number PRJNA690955, [https://www.ncbi.nlm.nih.gov/bioproject/?term=PRJNA690955]; and Sequence Read Archive (SRA) with accession number PRJNA622652 [https://trace.ncbi.nlm.nih.gov/Traces/sra/?study=SRP255396].
