# Supplementary material for: Differing coronavirus genres alter shared host signaling pathways upon viral infection
Source: Sci Rep. 2022 Jun 13;12:9744. doi: 10.1038/s41598-022-13396-7 (PMC9189807; doi:10.1038/s41598-022-13396-7)
Supplement: Supplementary file 1 — Supplementary Information 1. [file 41598_2022_13396_MOESM1_ESM.pdf]

Supplementary Information

Figure S1. DEGs in HIEC cells infected with PDCoV **(a),** NHBE cells infected with SARS-CoV-2 **(b)** and IPEC cells infected with SADS-CoV **(c)** at 24 hpi in **Cytokine – Cytokine Receptor Interaction Pathway*.**

(a)

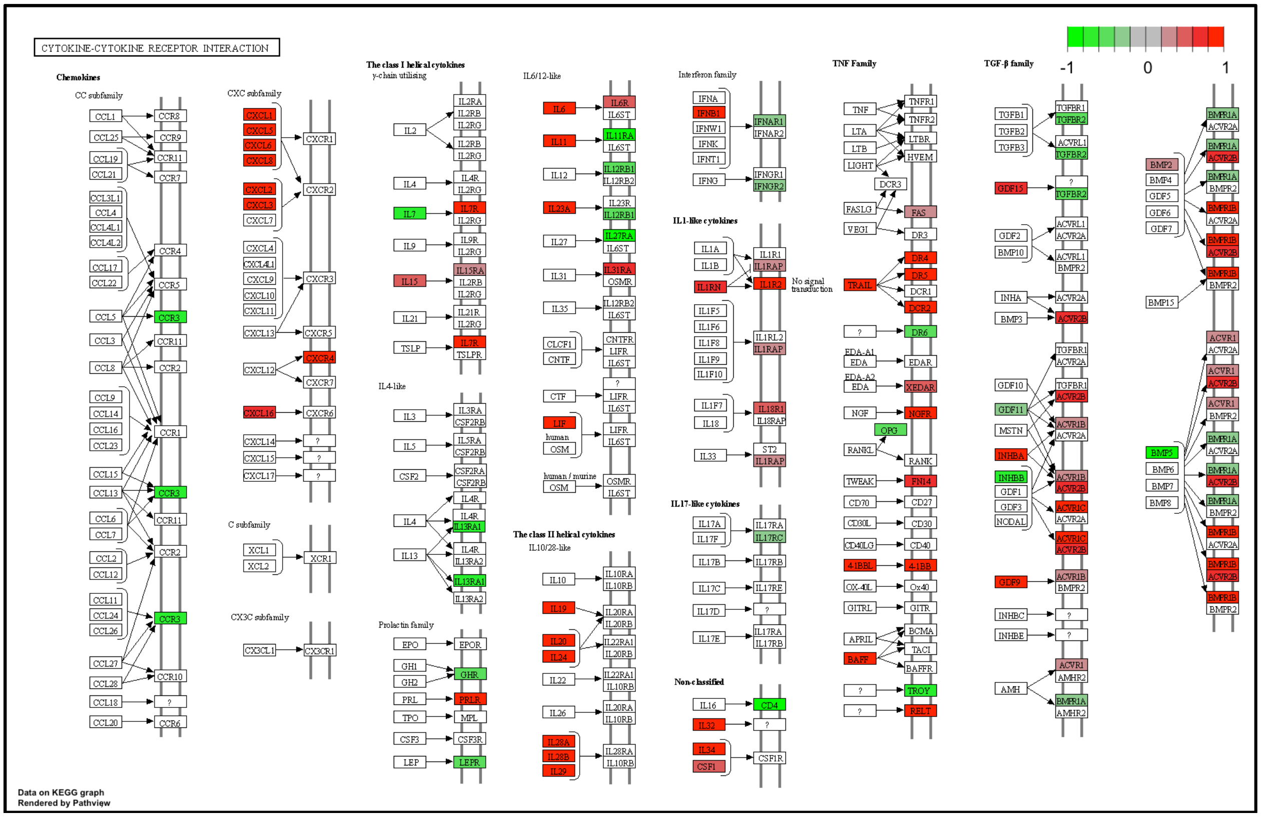

*Levels of increased and reduced expression of DE genes are shown in red and green, respectively.

(b)

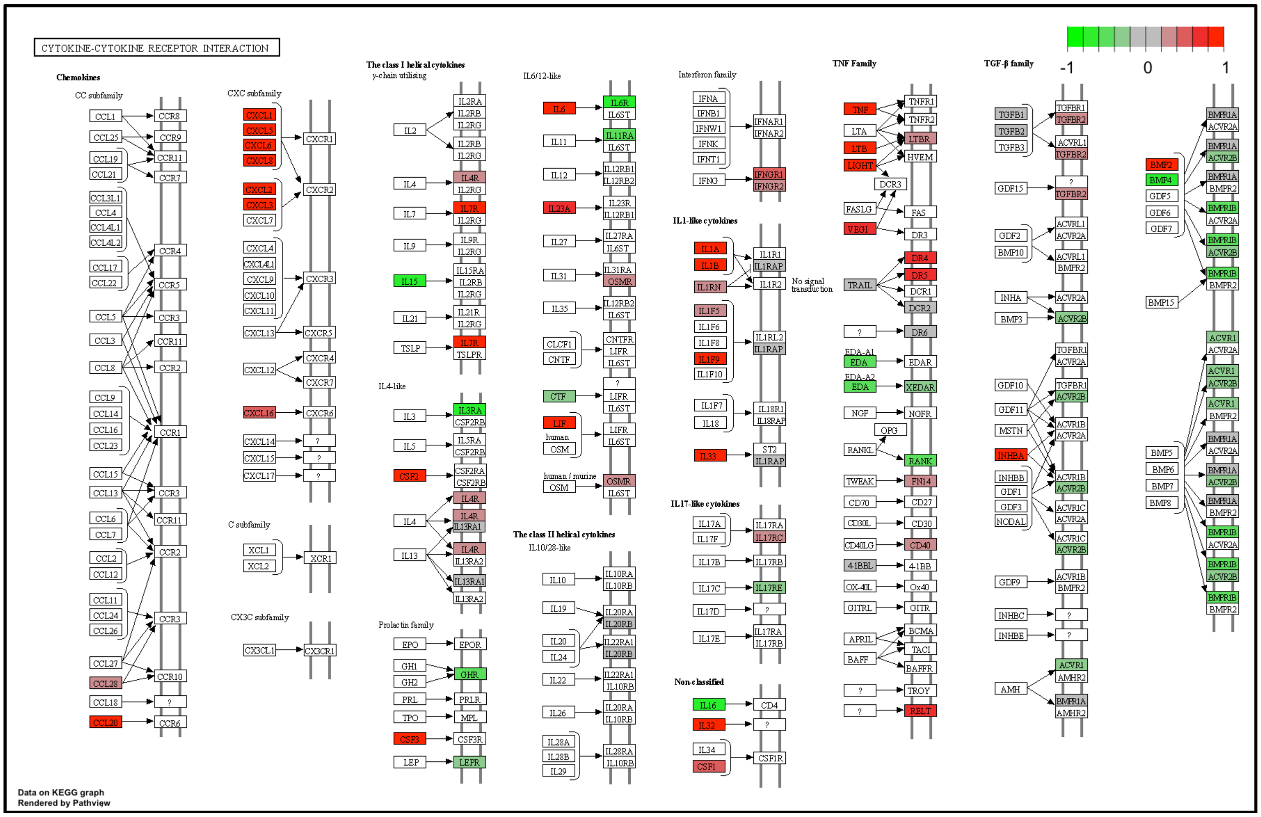

CYTOKINE-CYTOKINE RECEPTOR INTERACTION
Chemokines
CC subfamily
CXC subfamily
C subfamily
CX3C subfamily
The class I helical cytokines
γ-chain utilising
IL4-like
Prolactin family
IL6/12-like
The class II helical cytokines
IL10/28-like
Interferon family
IL1-like cytokines
No signal transduction
IL17-like cytokines
Non-classified
TNF Family
TGF-β family
-1
0
1
Data on KEGG graph
Rendered by Pathview

(c)

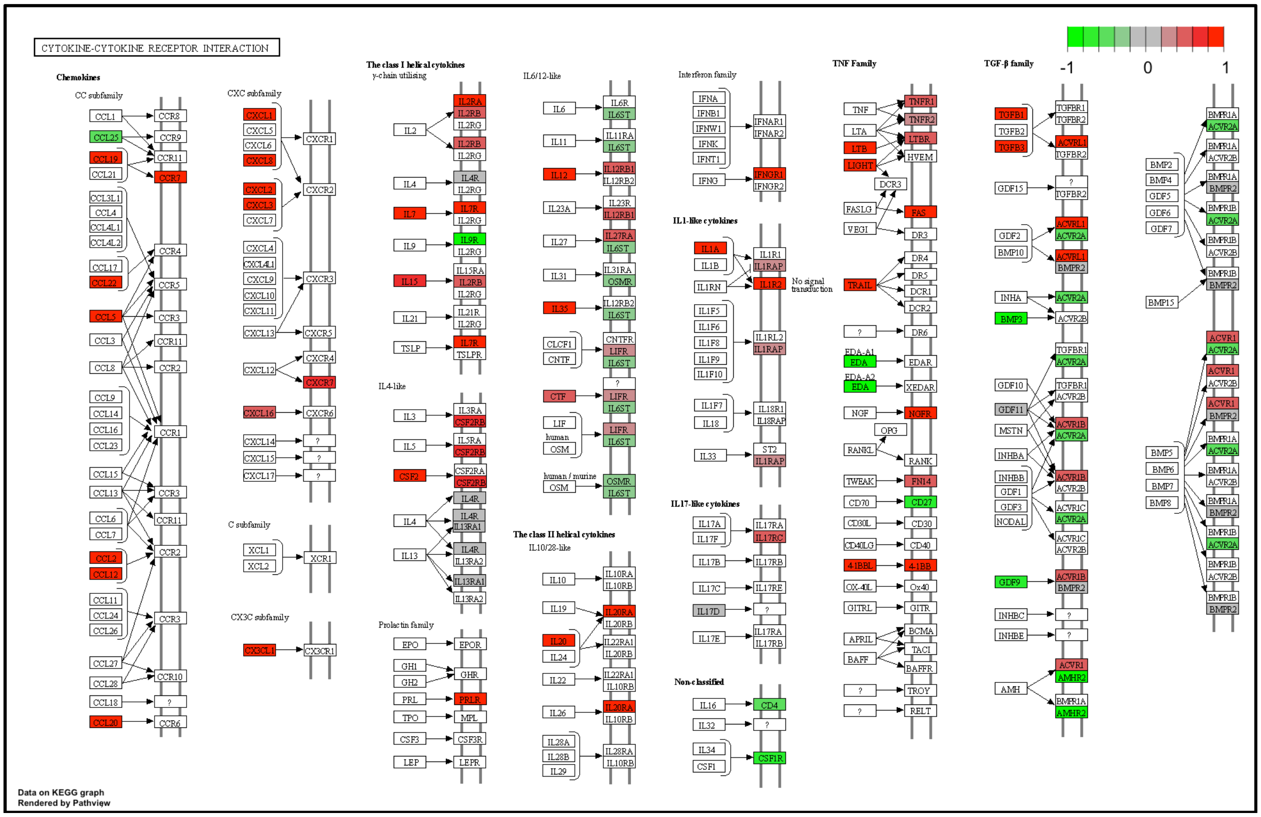

Figure S2. DEGs in HIEC cells infected with PDCoV **(a),** NHBE cells infected with SARS-CoV-2 **(b)** and IPEC cells infected with SADS-CoV **(c)** at 24 hpi in **Viral protein Interaction Pathway with cytokine and cytokine receptor.**

(a)

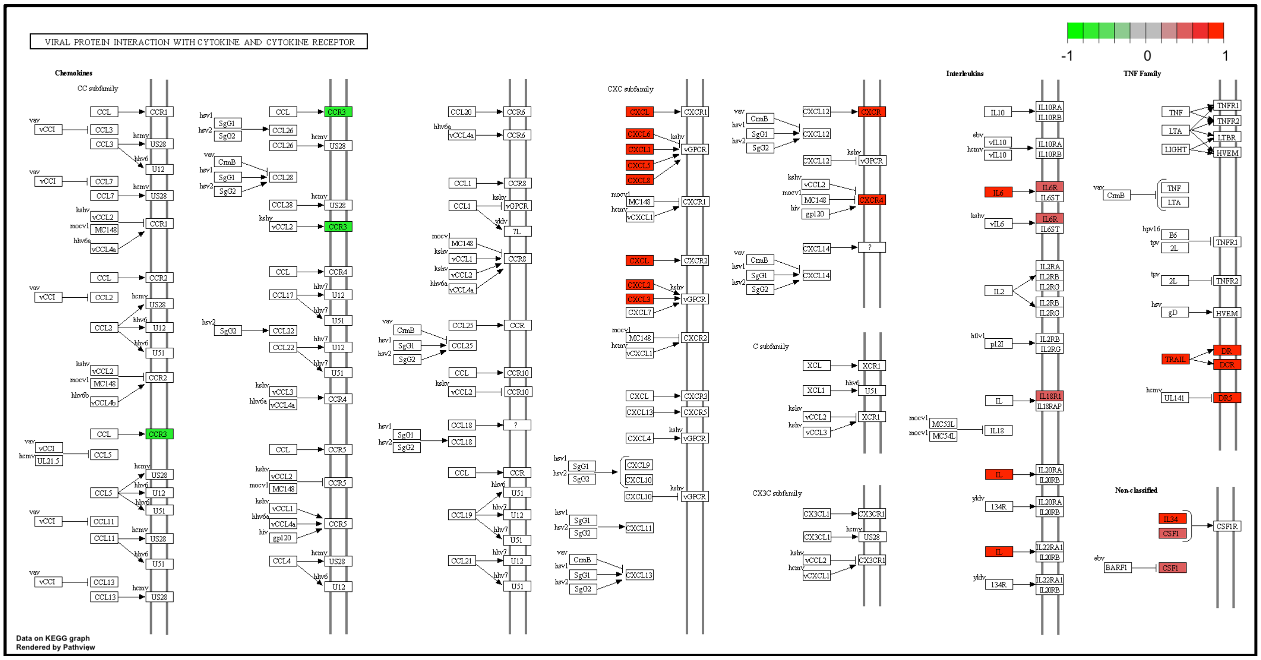

(b)

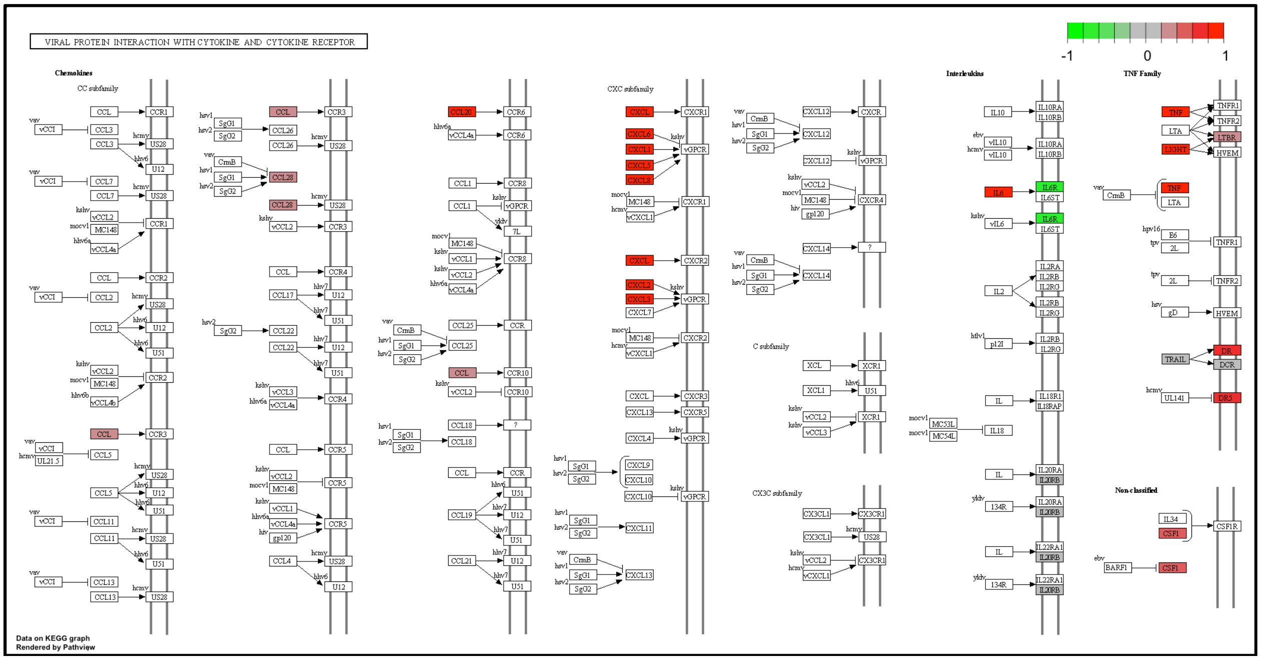

(c)

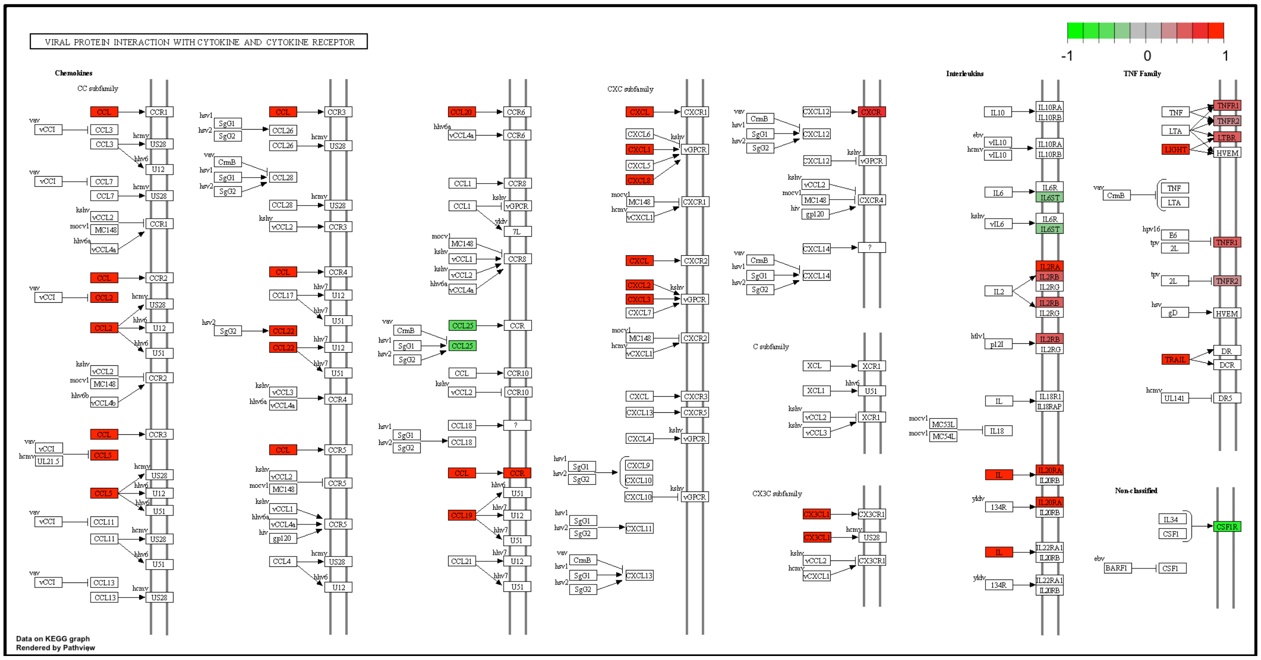

Figure S3. DEGs in HIEC cells infected with PDCoV **(a),** NHBE cells infected with SARS-CoV-2 **(b)** and IPEC cells infected with SADS-CoV **(c)** at 24 hpi in **NF-Kappa B signaling pathway.**

(a)

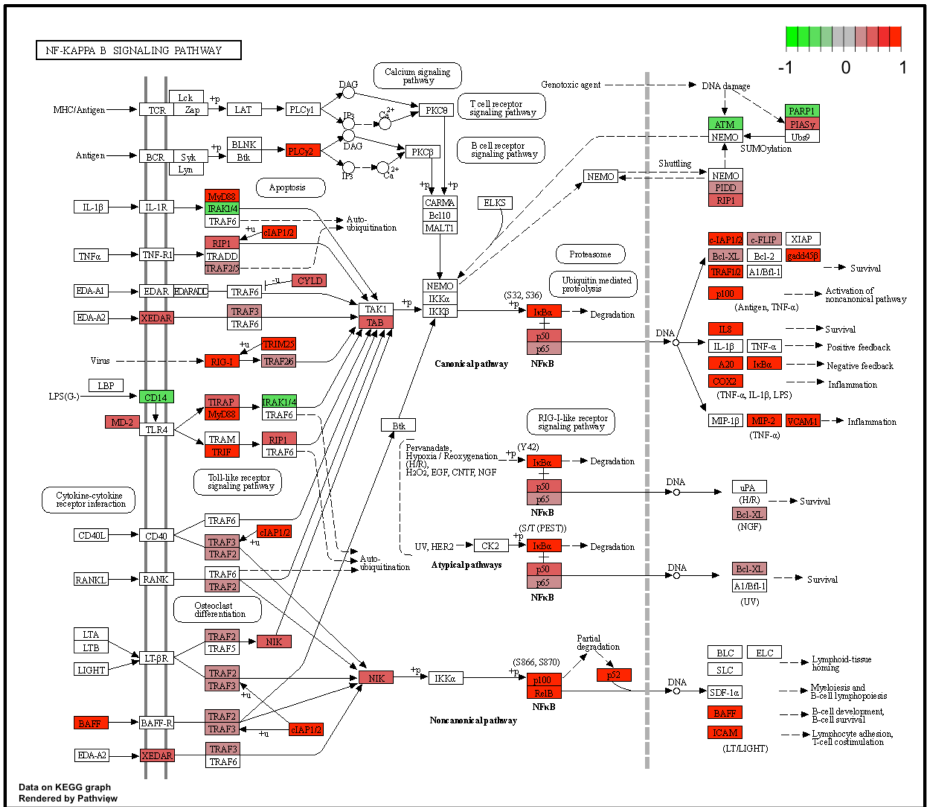
NF-KAPPA B SIGNALING PATHWAY
-1
0
1
MHC/Antigen
Antigen
TCR
BCR
Lck
Zap
Syk
Lyn
LAT
BLNK
Btk
PLCγ1
PLCγ2
DAG
IP3
Ca2+
PKCθ
PKCβ
Calcium signaling pathway
T cell receptor signaling pathway
B cell receptor signaling pathway
Apoptosis
CARMA
Bcl10
MALT1
ELKS
IL-1β
IL-1R
MyD88
IRAK1/4
TRAF6
cIAP1/2
Auto-ubiquitination
TNFα
TNF-R1
RIP1
TRADD
TRAF2/5
EDA-A1
EDAR
EDARADD
CYLD
EDA-A2
XEDAR
TRAF3
TAK1
TAB
NEMO
IKKα
IKKβ
Virus
RIG-I
TRIM25
TRAF2/6
LBP
LPS(G-)
CD14
MD-2
TLR4
TIRAP
TRAM
TRIF
Toll-like receptor signaling pathway
Cytokine-cytokine receptor interaction
CD40L
CD40
RANKL
RANK
Osteoclast differentiation
LTA
LTB
LIGHT
LT-βR
TRAF5
NIK
BAFF
BAFF-R
Btk
Genotoxic agent
DNA damage
ATM
PARP1
PIASy
Ubc9
SUMOylation
Shuttling
PIDD
Proteasome
Ubiquitin mediated proteolysis
(S32, S36)
IκBα
p50
p65
NFκB
Degradation
Canonical pathway
DNA
c-IAP1/2
c-FLIP
XIAP
Bcl-XL
Bcl-2
gadd45β
TRAF1/2
A1/Bfl-1
Survival
p100
Activation of noncanonical pathway
(Antigen, TNF-α)
IL8
IL-1β
TNF-α
A20
COX2
Positive feedback
Negative feedback
Inflammation
(TNF-α, IL-1β, LPS)
MIP-1β
MIP-2
VCAM-1
RIG-I-like receptor signaling pathway
Pervanadate, Hypoxia / Reoxygenation (H/R), H2O2, EGF, CNTF, NGF
(Y42)
uPA
(H/R)
(NGF)
UV, HER2
CK2
(S/T (PEST))
Atypical pathways
(UV)
Partial degradation
(S866, S870)
RelB
p52
Noncanonical pathway
BLC
ELC
SLC
SDF-1α
ICAM
(LT/LIGHT)
Lymphoid-tissue homing
Myeloiesis and B-cell lymphopoiesis
B-cell development, B-cell survival
Lymphocyte adhesion, T-cell costimulation
Data on KEGG graph
Rendered by Pathview

(b)

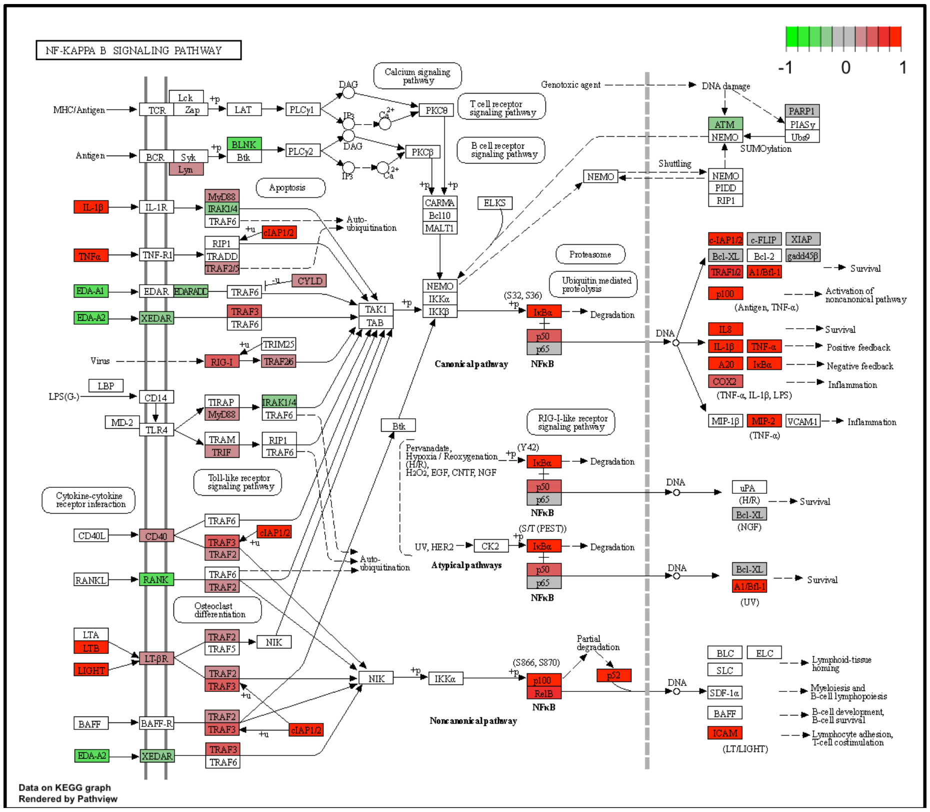

(c)

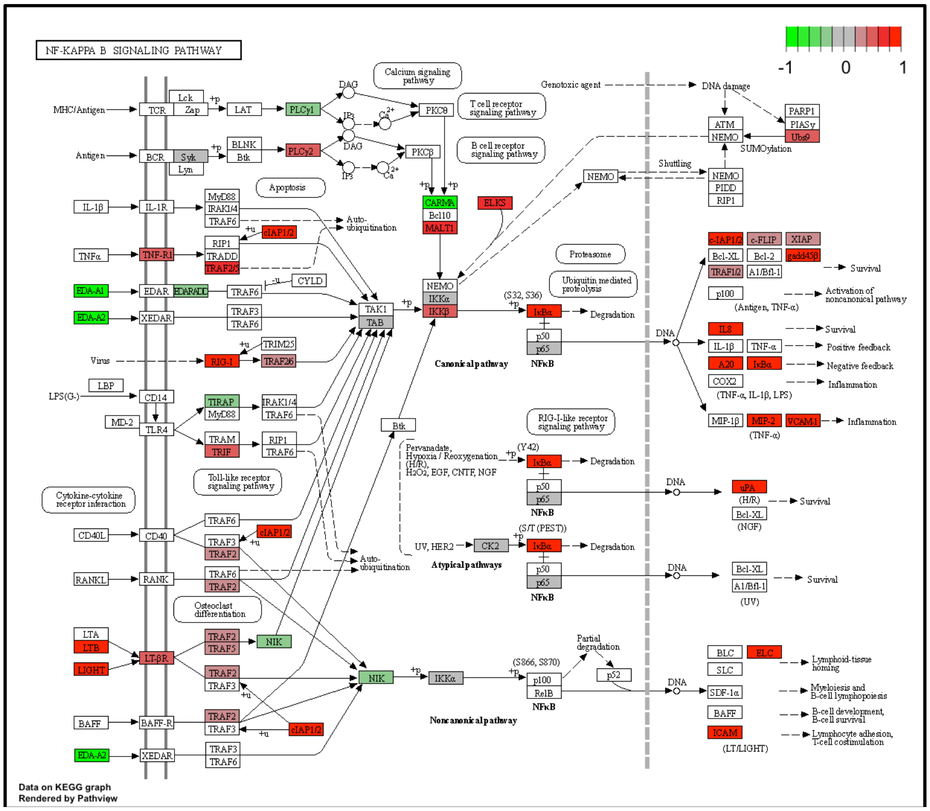

Figure S4. DEGs in HIEC cells infected with PDCoV **(a)**, NHBE cells infected with SARS-CoV-2 **(b)** and IPEC cells infected with SADS-CoV **(c)** at 24 hpi in **Toll like receptor signaling pathway.**

(a)

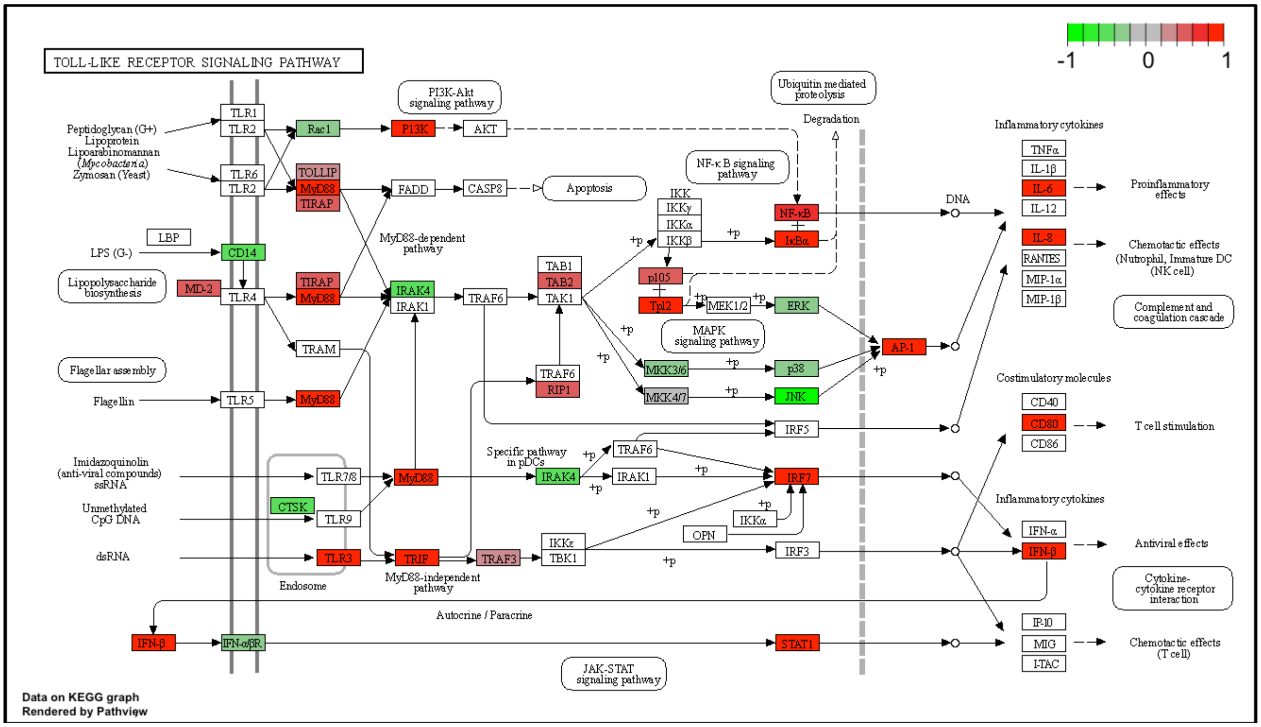

(b)

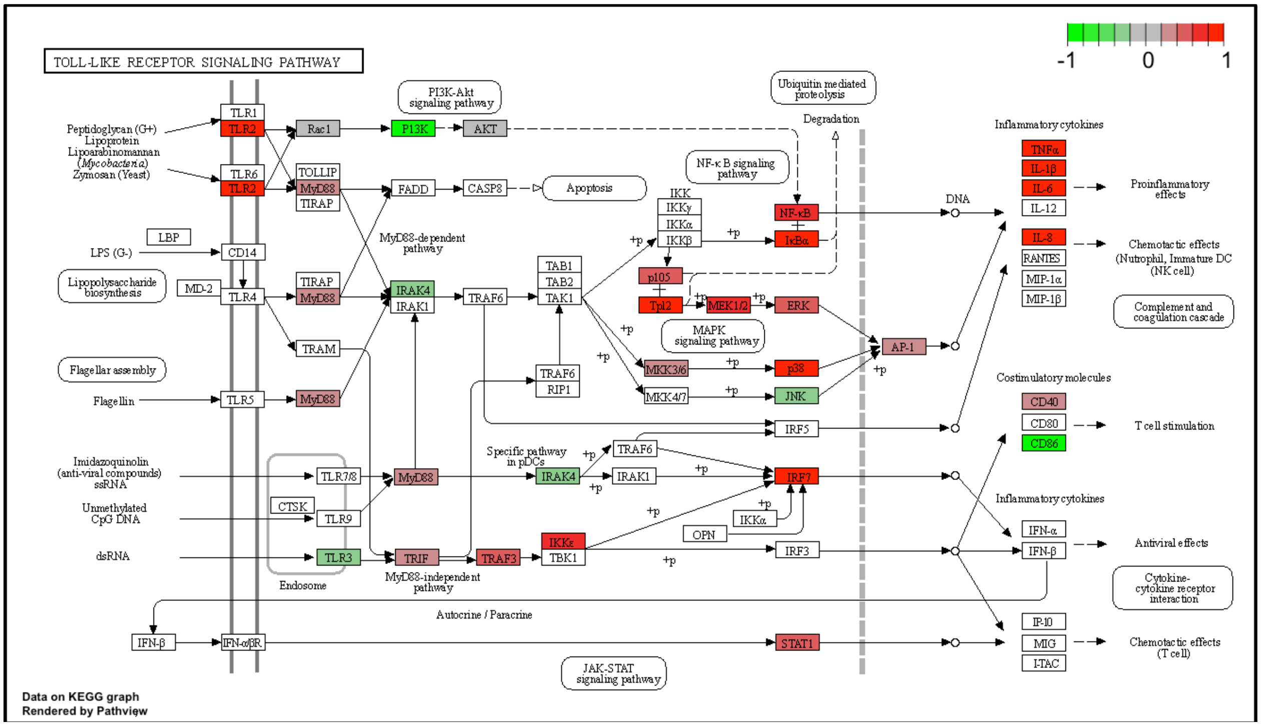

(c)

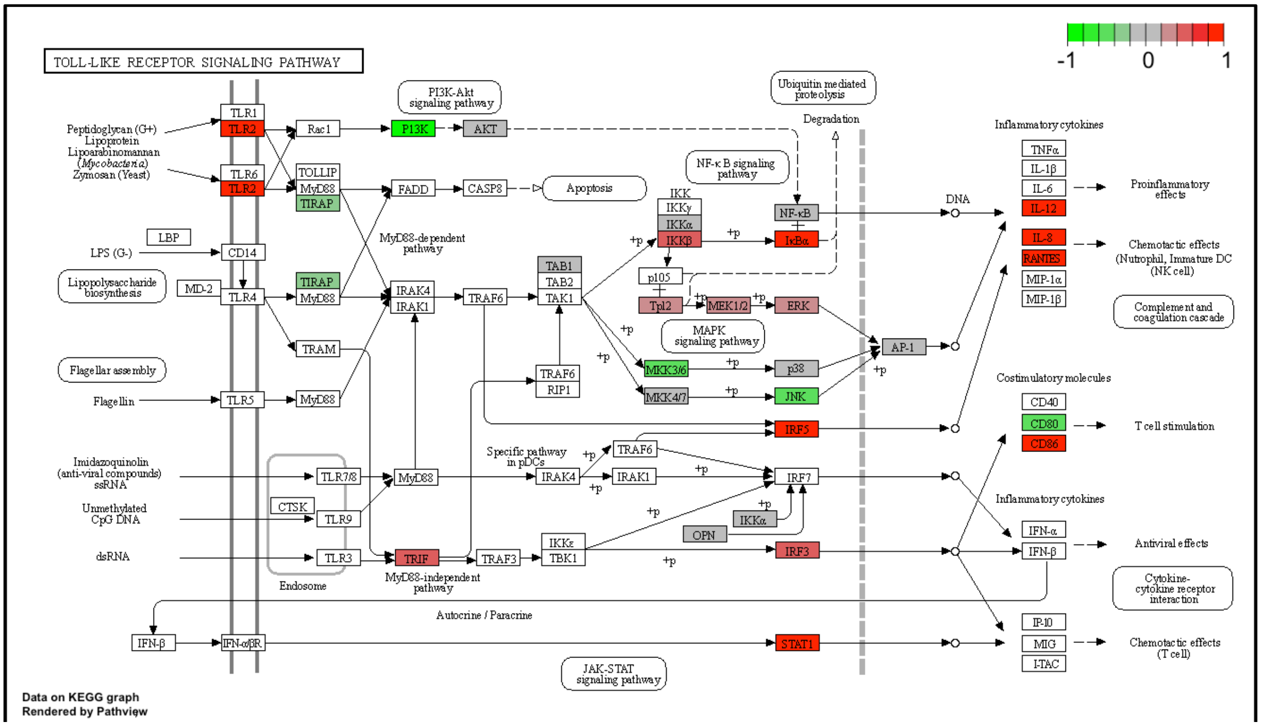

Figure S5. DEGs in HIEC cells infected with PDCoV **(a),** NHBE cells infected with SARS-CoV-2 **(b)** and IPEC cells infected with SADS-CoV **(c)** at 24 hpi in **NOD like receptor signaling pathway.**

(a)

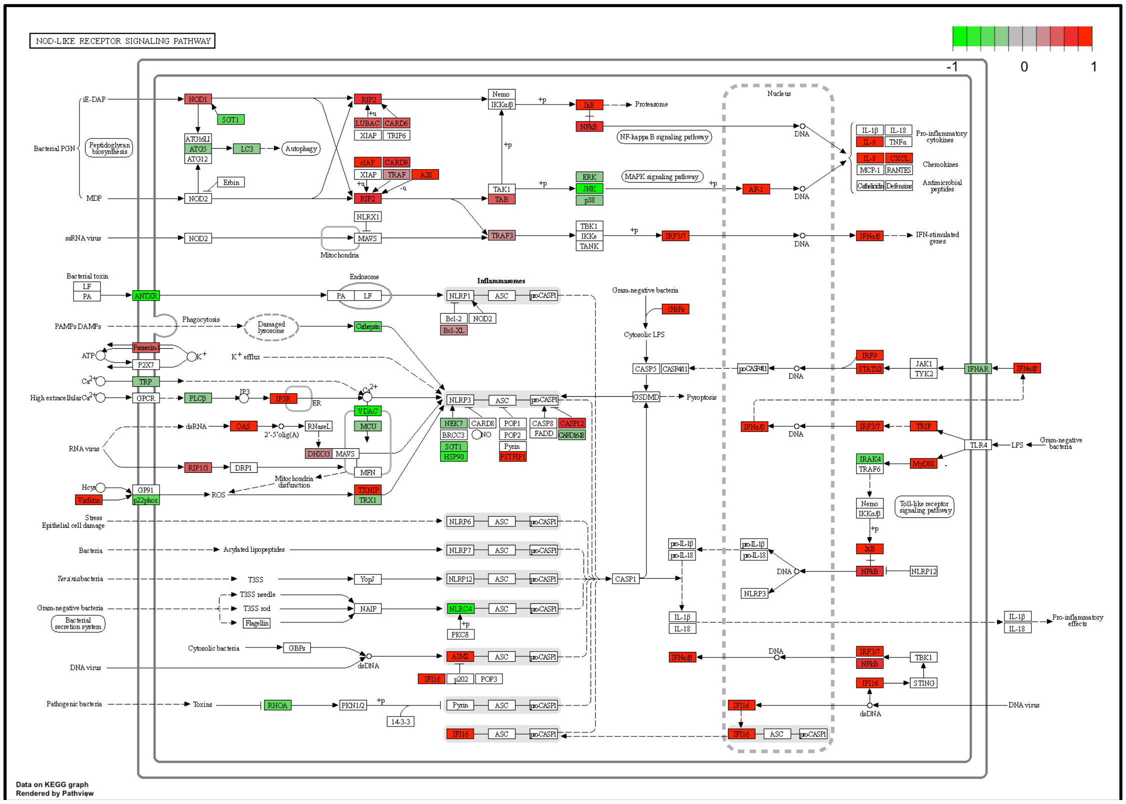
NOD-LIKE RECEPTOR SIGNALING PATHWAY
-1
0
1
Data on KEGG graph
Rendered by Pathview

(b)

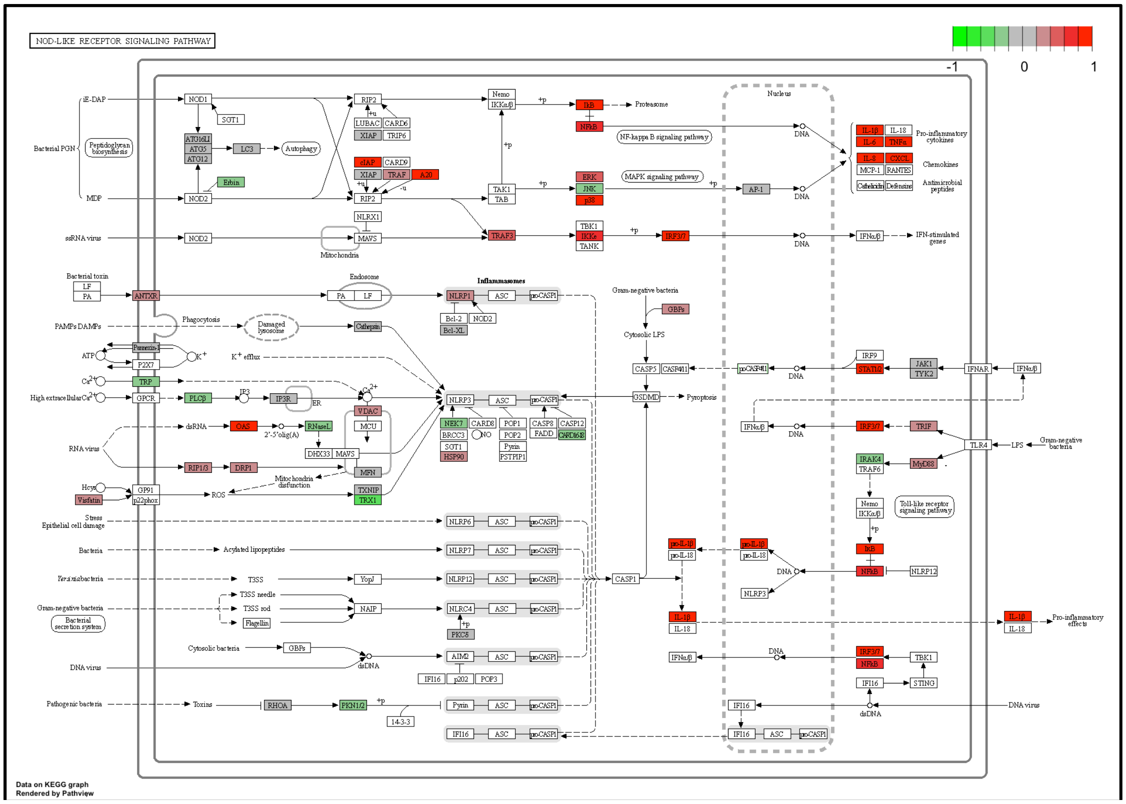
NOD-LIKE RECEPTOR SIGNALING PATHWAY
-1
0
1
Data on KEGG graph
Rendered by Pathview

(c)

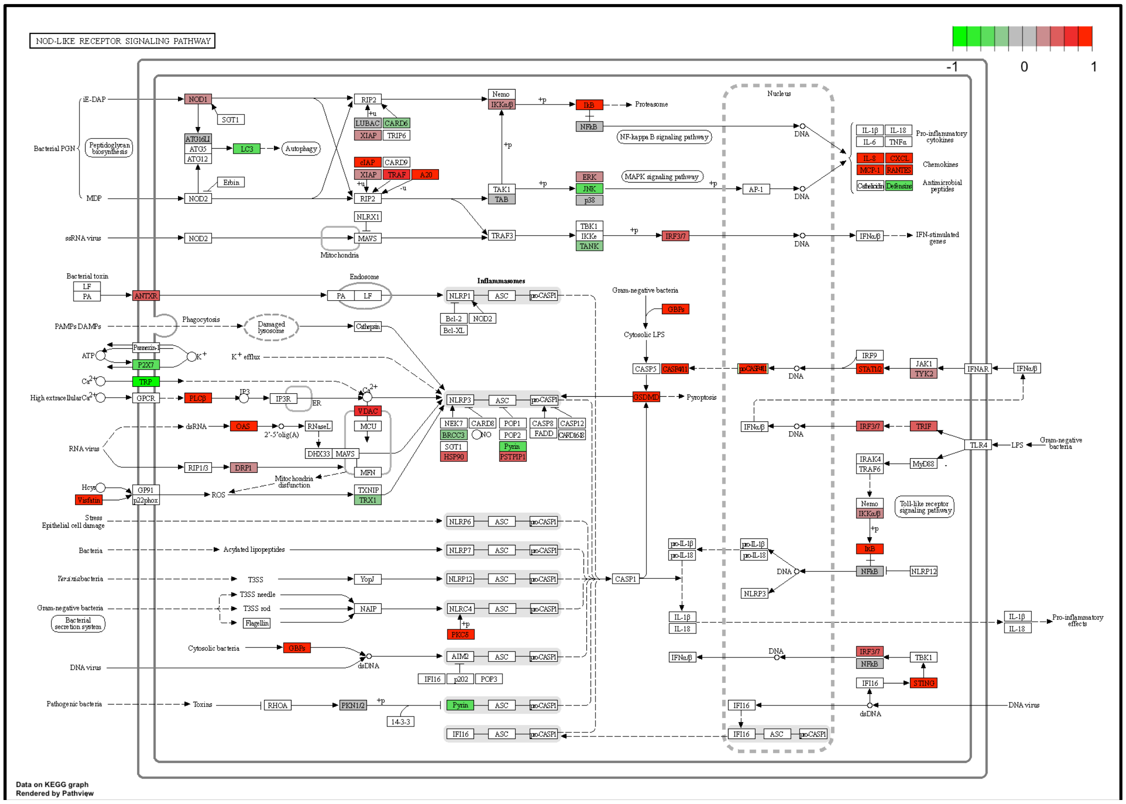

Figure S6. DEGs in HIEC cells infected with PDCoV **(a),** NHBE cells infected with SARS-CoV-2 **(b)** and IPEC cells infected with SADS-CoV **(c)** at 24 hpi in **RIG -I like receptor signaling pathway.**

(a)

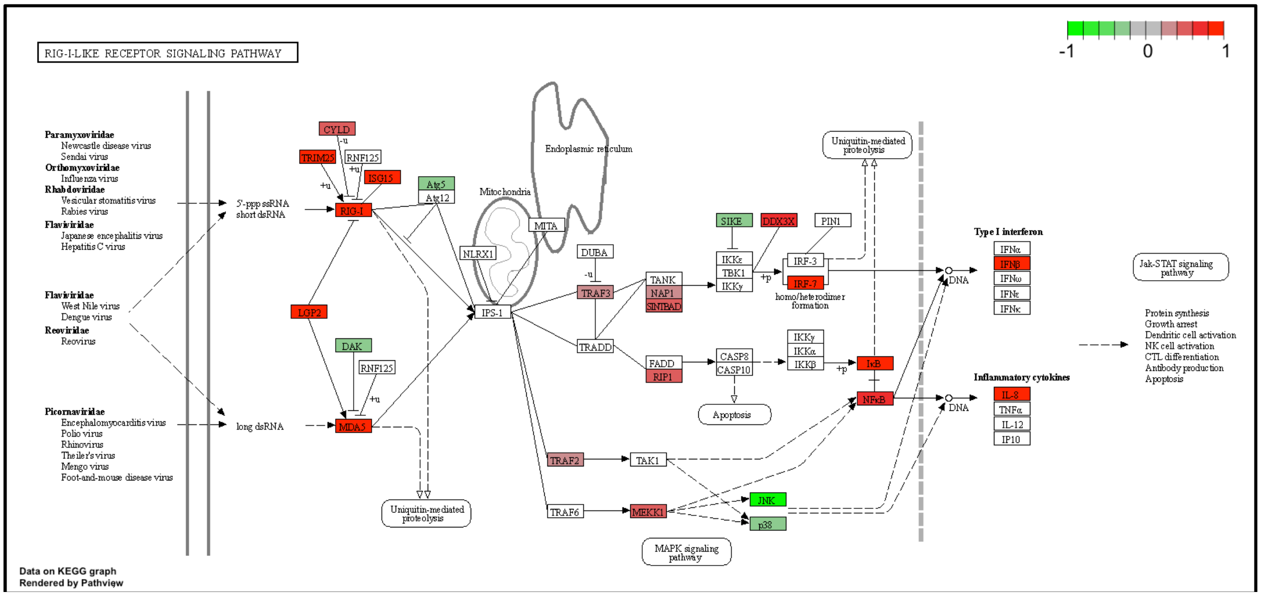

(b)

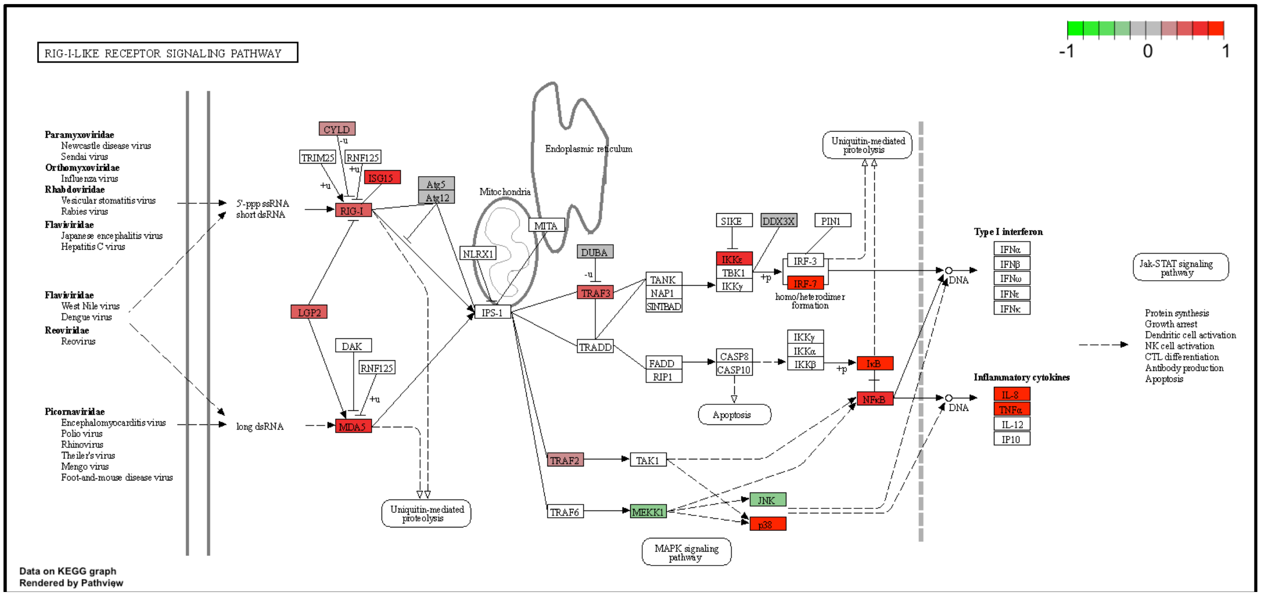

(c)

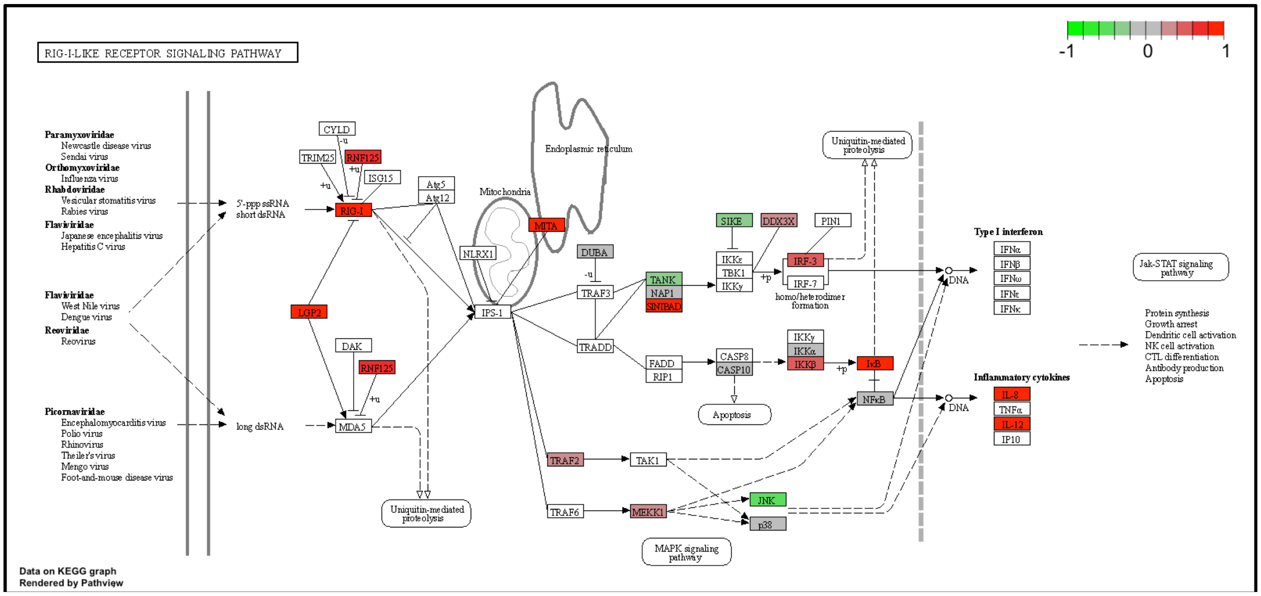

Figure S7. DEGs in HIEC cells infected with PDCoV **(a),** NHBE cells infected with SARS-CoV-2 **(b)** and IPEC cells infected with SADS-CoV **(c)** at 24 hpi in **Cytosolic DNA signaling pathway.**

(a)

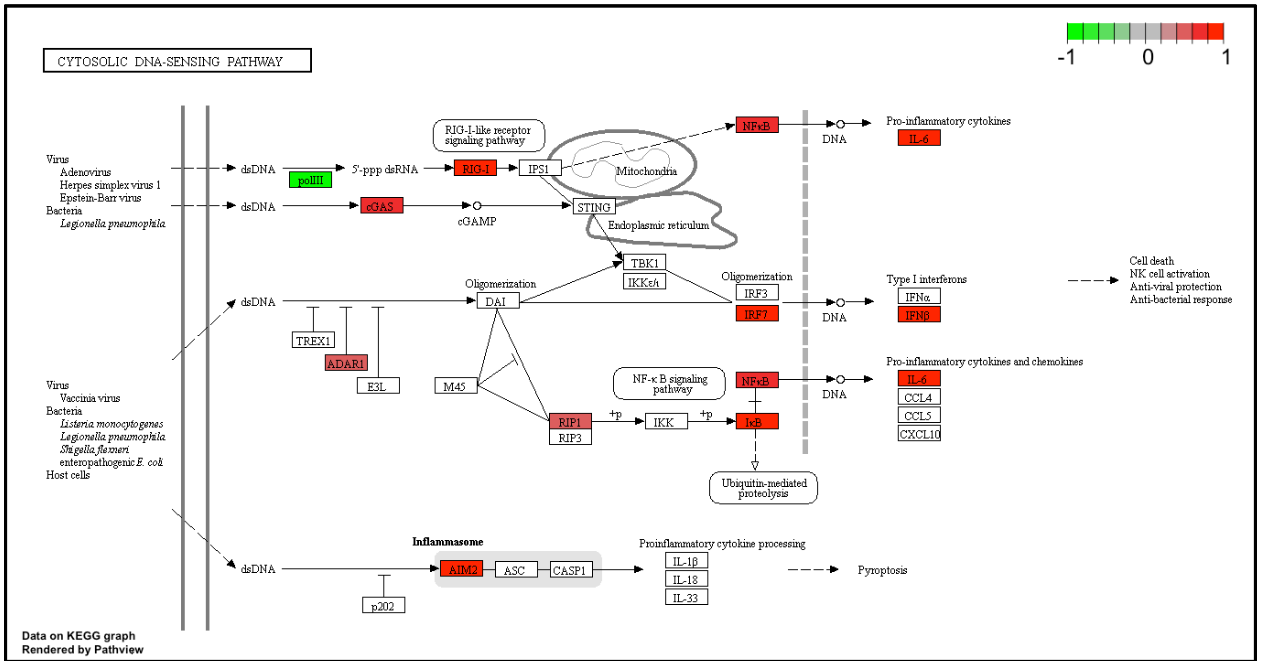
CYTOSOLIC DNA-SENSING PATHWAY
-1
0
1
Virus
Adenovirus
Herpes simplex virus 1
Epstein-Barr virus
Bacteria
Legionella pneumophila
dsDNA
polIII
5'-ppp dsRNA
RIG-I-like receptor signaling pathway
RIG-I
IPS1
Mitochondria
NFκB
DNA
Pro-inflammatory cytokines
IL-6
cGAS
cGAMP
STING
Endoplasmic reticulum
TBK1
IKKε/i
Oligomerization
IRF3
IRF7
Type I interferons
IFNα
IFNβ
Cell death
NK cell activation
Anti-viral protection
Anti-bacterial response
DAI
TREX1
ADAR1
E3L
M45
Virus
Vaccinia virus
Bacteria
Listeria monocytogenes
Legionella pneumophila
Shigella flexneri
enteropathogenic E. coli
Host cells
NF-κB signaling pathway
RIP1
RIP3
+p
IKK
IκB
Pro-inflammatory cytokines and chemokines
CCL4
CCL5
CXCL10
Ubiquitin-mediated proteolysis
Inflammasome
AIM2
ASC
CASP1
p202
Proinflammatory cytokine processing
IL-1β
IL-18
IL-33
Pyroptosis
Data on KEGG graph
Rendered by Pathview

(b)

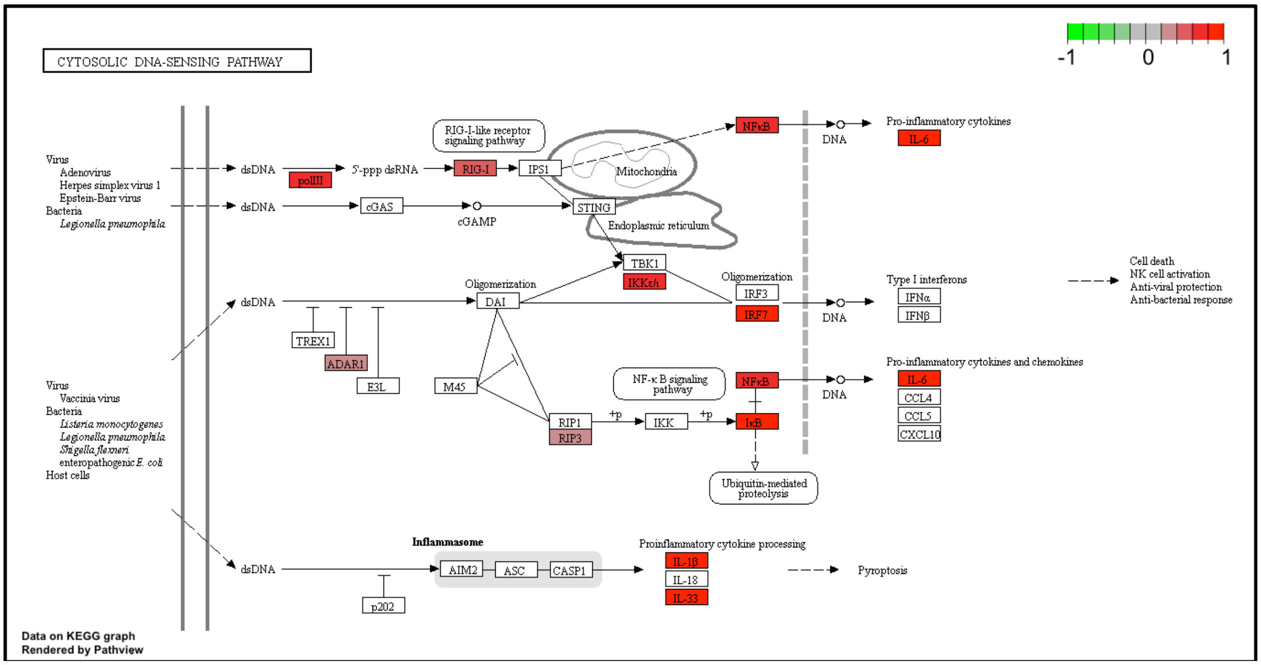
CYTOSOLIC DNA-SENSING PATHWAY
-1
0
1
Virus
Adenovirus
Herpes simplex virus 1
Epstein-Barr virus
Bacteria
Legionella pneumophila
dsDNA
polIII
5'-ppp dsRNA
RIG-I-like receptor signaling pathway
RIG-I
IPS1
Mitochondria
NFκB
DNA
Pro-inflammatory cytokines
IL-6
cGAS
cGAMP
STING
Endoplasmic reticulum
TBK1
IKKε/i
Oligomerization
IRF3
IRF7
Type I interferons
IFNα
IFNβ
Cell death
NK cell activation
Anti-viral protection
Anti-bacterial response
DAI
TREX1
ADAR1
E3L
M45
Virus
Vaccinia virus
Bacteria
Listeria monocytogenes
Legionella pneumophila
Shigella flexneri
enteropathogenic E. coli
Host cells
NF-κB signaling pathway
RIP1
RIP3
+p
IKK
IκB
Pro-inflammatory cytokines and chemokines
CCL4
CCL5
CXCL10
Ubiquitin-mediated proteolysis
Inflammasome
AIM2
ASC
CASP1
p202
Proinflammatory cytokine processing
IL-1β
IL-18
IL-33
Pyroptosis
Data on KEGG graph
Rendered by Pathview

(c)

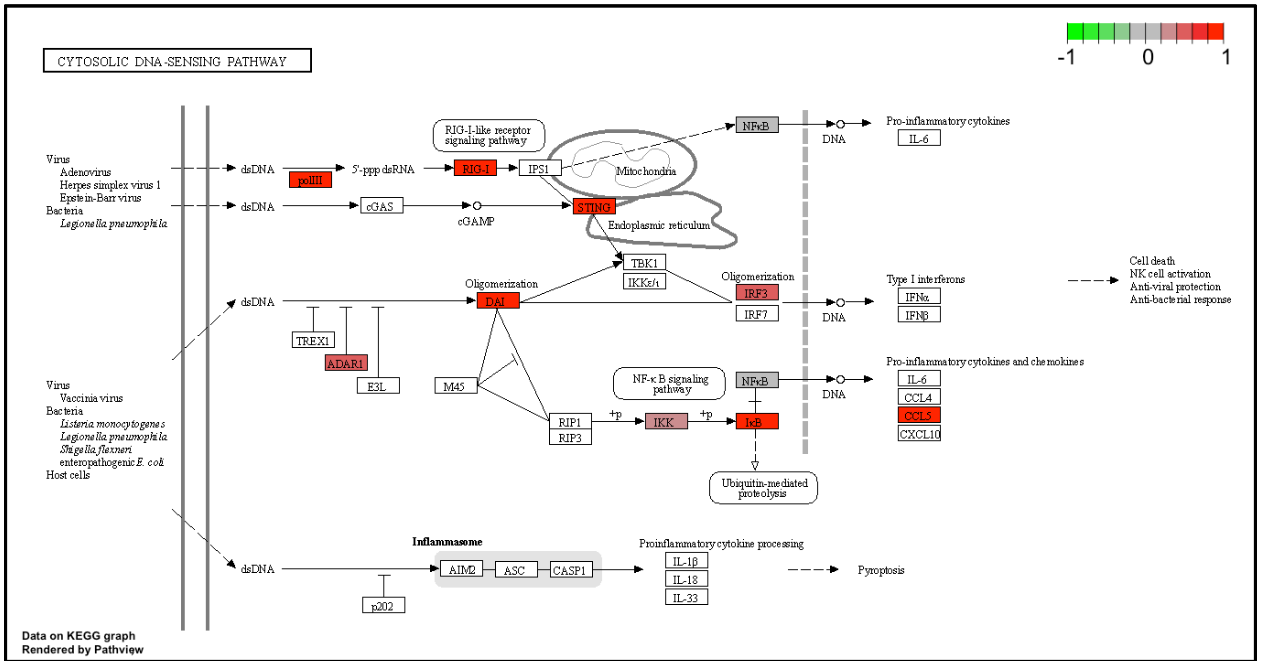

Figure S8. DEGs in HIEC cells infected with PDCoV **(a)**, NHBE cells infected with SARS-CoV-2 **(b)** and IPEC cells infected with SADS-CoV **(c)** at 24 hpi in **JAK-STAT signaling pathway.**

(a)

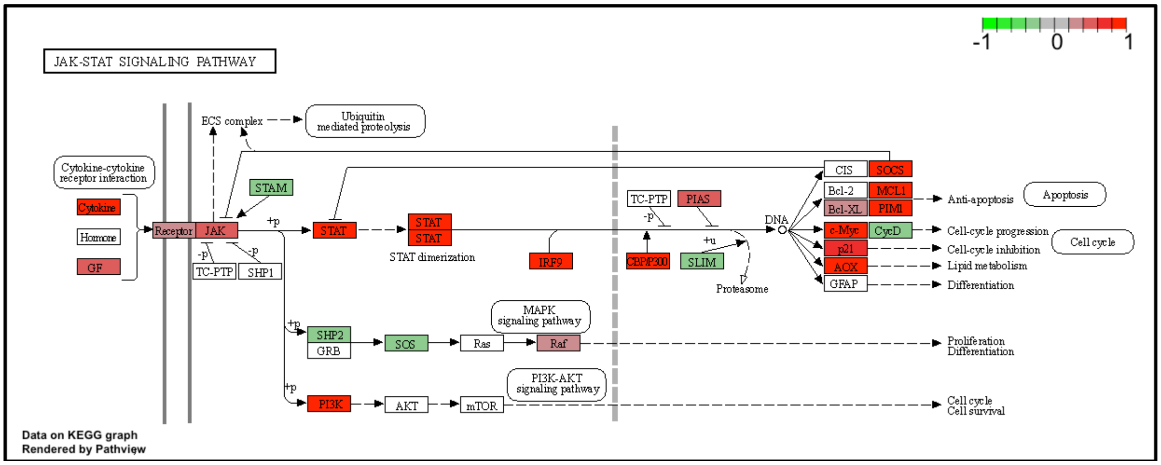

(b)

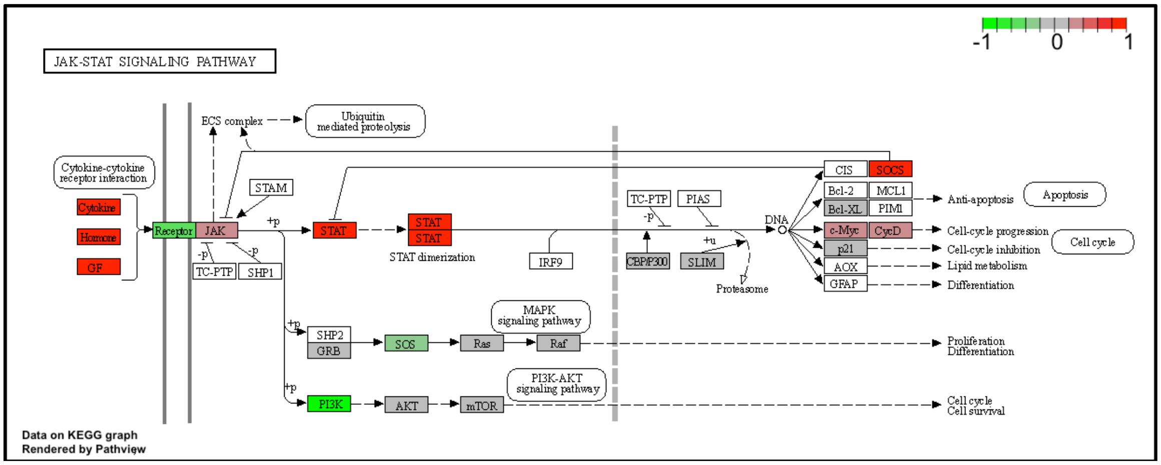

(c)

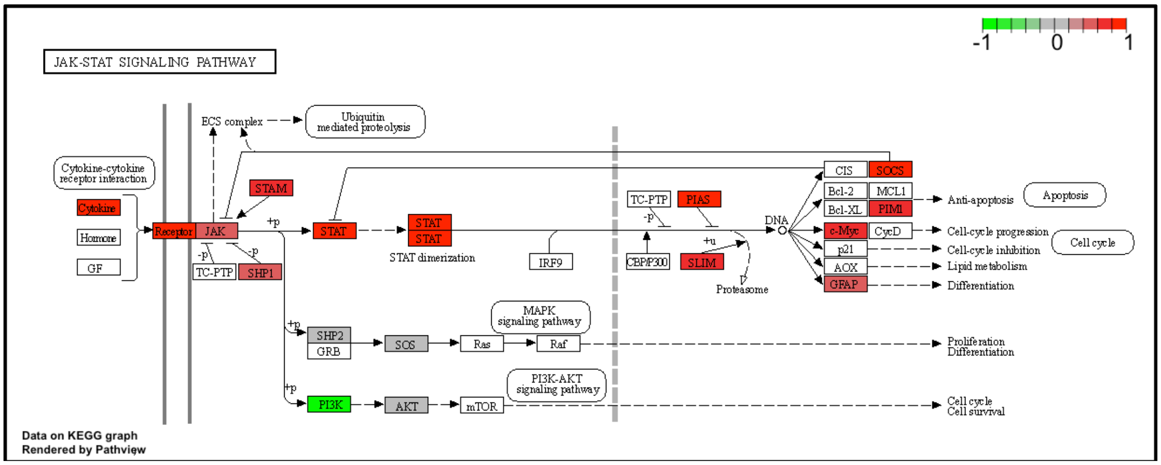

Figure S9. DEGs in HIEC cells infected with PDCoV **(a),** NHBE cells infected with SARS-CoV-2 **(b)** and IPEC cells infected with SADS-CoV **(c)** at 24 hpi in **IL-17 signaling pathway.**

(a)

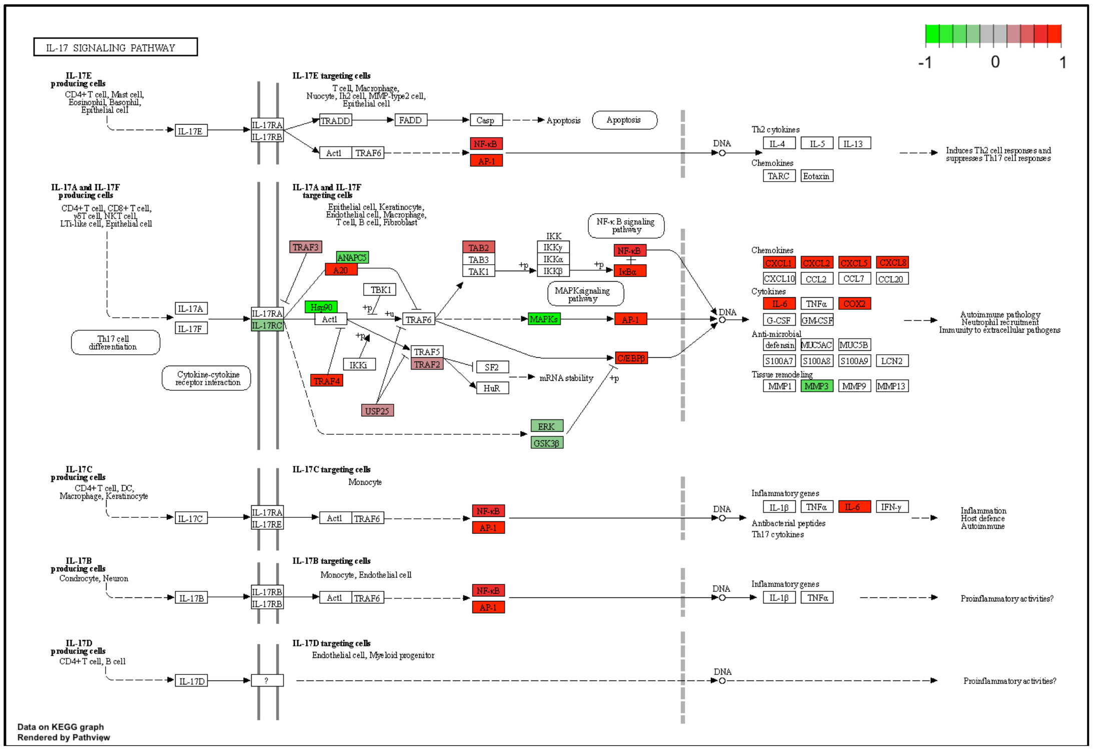
IL-17 SIGNALING PATHWAY
-1
0
1
IL-17E producing cells
CD4+ T cell, Mast cell, Eosinophil, Basophil, Epithelial cell
IL-17E
IL-17RA
IL-17RB
IL-17E targeting cells
T cell, Macrophage, Nuocyte, Ih2 cell, MMP-type2 cell, Epithelial cell
TRADD
FADD
Casp
Apoptosis
Act1
TRAF6
NF-κB
AP-1
DNA
Th2 cytokines
IL-4
IL-5
IL-13
Chemokines
TARC
Eotaxin
Induces Th2 cell responses and suppresses Th17 cell responses
IL-17A and IL-17F producing cells
CD4+ T cell, CD8+ T cell, γδT cell, NKT cell, LTi-like cell, Epithelial cell
IL-17A
IL-17F
IL-17RC
Th17 cell differentiation
Cytokine-cytokine receptor interaction
IL-17A and IL-17F targeting cells
Epithelial cell, Keratinocyte, Endothelial cell, Macrophage, T cell, B cell, Fibroblast
TRAF3
ANAPC5
A20
Hsp90
TBK1
IKKi
TRAF4
USP25
TRAF5
TRAF2
SF2
HuR
mRNA stability
TAB2
TAB3
TAK1
IKK
IKKγ
IKKα
IKKβ
NF-κB signaling pathway
MAPK signaling pathway
IκBα
MAPKs
C/EBPβ
ERK
GSK3β
+p
+u
Chemokines
CXCL1
CXCL2
CXCL5
CXCL8
CXCL10
CCL2
CCL7
CCL20
Cytokines
IL-6
TNFα
COX2
G-CSF
GM-CSF
Anti-microbial
defensin
MUC5AC
MUC5B
S100A7
S100A8
S100A9
LCN2
Tissue remodeling
MMP1
MMP3
MMP9
MMP13
Autoimmune pathology
Neutrophil recruitment
Immunity to extracellular pathogens
IL-17C producing cells
CD4+ T cell, DC, Macrophage, Keratinocyte
IL-17C
IL-17RE
IL-17C targeting cells
Monocyte
Inflammatory genes
IL-1β
IFN-γ
Antibacterial peptides
Th17 cytokines
Inflammation
Host defence
Autoimmune
IL-17B producing cells
Condrocyte, Neuron
IL-17B
IL-17B targeting cells
Monocyte, Endothelial cell
Proinflammatory activities?
IL-17D producing cells
CD4+ T cell, B cell
IL-17D
?
IL-17D targeting cells
Endothelial cell, Myeloid progenitor
Data on KEGG graph
Rendered by Pathview

(b)

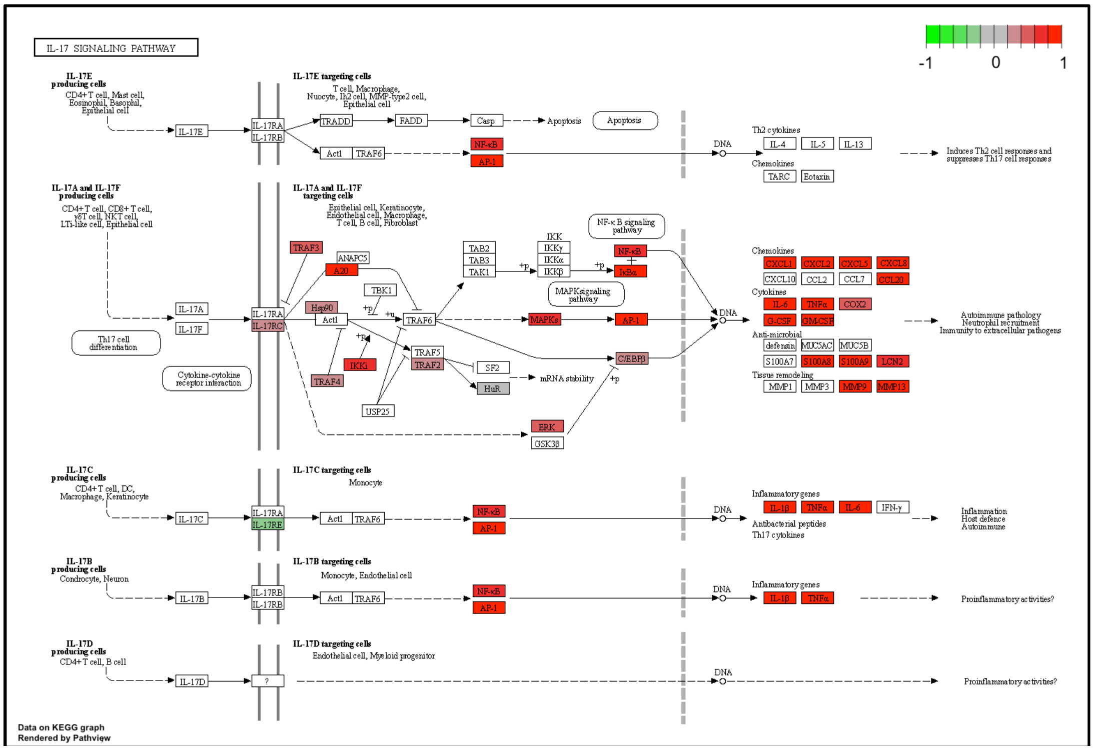
IL-17 SIGNALING PATHWAY
-1
0
1
IL-17E producing cells
CD4+ T cell, Mast cell, Eosinophil, Basophil, Epithelial cell
IL-17E
IL-17RA
IL-17RB
IL-17E targeting cells
T cell, Macrophage, Nuocyte, Ih2 cell, MMP-type2 cell, Epithelial cell
TRADD
FADD
Casp
Apoptosis
Act1
TRAF6
NF-κB
AP-1
DNA
Th2 cytokines
IL-4
IL-5
IL-13
Chemokines
TARC
Eotaxin
Induces Th2 cell responses and suppresses Th17 cell responses
IL-17A and IL-17F producing cells
CD4+ T cell, CD8+ T cell, γδT cell, NKT cell, LTi-like cell, Epithelial cell
IL-17A
IL-17F
IL-17RC
Th17 cell differentiation
Cytokine-cytokine receptor interaction
IL-17A and IL-17F targeting cells
Epithelial cell, Keratinocyte, Endothelial cell, Macrophage, T cell, B cell, Fibroblast
TRAF3
ANAPC5
A20
Hsp90
TBK1
IKKi
TRAF4
USP25
TRAF5
TRAF2
SF2
HuR
mRNA stability
TAB2
TAB3
TAK1
IKK
IKKγ
IKKα
IKKβ
NF-κB signaling pathway
MAPK signaling pathway
IκBα
MAPKs
C/EBPβ
ERK
GSK3β
+p
+u
Chemokines
CXCL1
CXCL2
CXCL5
CXCL8
CXCL10
CCL2
CCL7
CCL20
Cytokines
IL-6
TNFα
COX2
G-CSF
GM-CSF
Anti-microbial
defensin
MUC5AC
MUC5B
S100A7
S100A8
S100A9
LCN2
Tissue remodeling
MMP1
MMP3
MMP9
MMP13
Autoimmune pathology
Neutrophil recruitment
Immunity to extracellular pathogens
IL-17C producing cells
CD4+ T cell, DC, Macrophage, Keratinocyte
IL-17C
IL-17RE
IL-17C targeting cells
Monocyte
Inflammatory genes
IL-1β
IFN-γ
Antibacterial peptides
Th17 cytokines
Inflammation
Host defence
Autoimmune
IL-17B producing cells
Condrocyte, Neuron
IL-17B
IL-17B targeting cells
Monocyte, Endothelial cell
Proinflammatory activities?
IL-17D producing cells
CD4+ T cell, B cell
IL-17D
?
IL-17D targeting cells
Endothelial cell, Myeloid progenitor
Data on KEGG graph
Rendered by Pathview

(c)

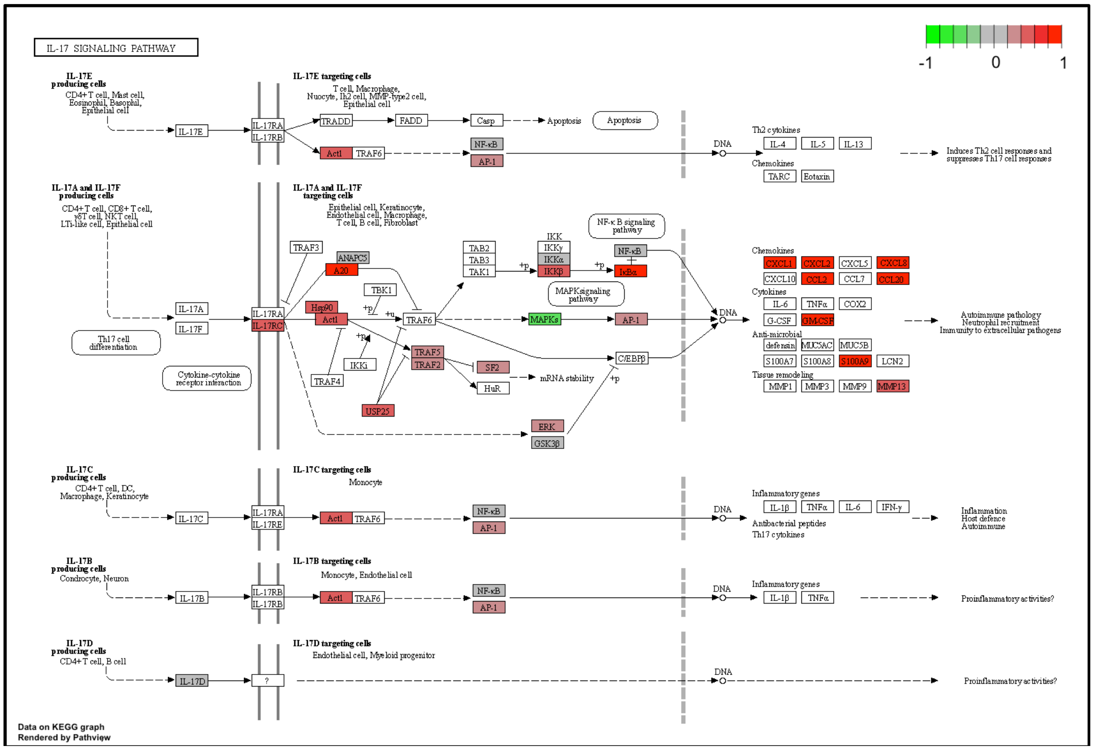

Figure S10. DEGs in HIEC cells infected with PDCoV **(a),** NHBE cells infected with SARS-CoV-2 **(b)** and IPEC cells infected with SADS-CoV **(c)** at 24 hpi in **TNF signaling pathway.**

(a)

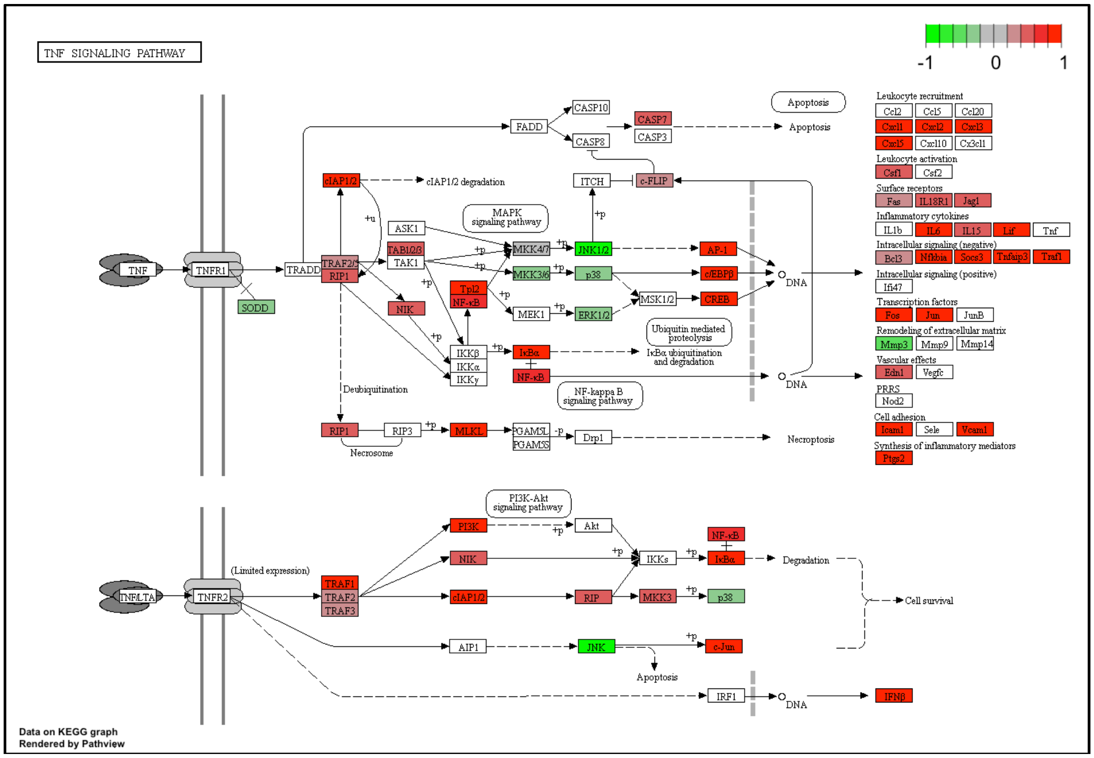

(b)

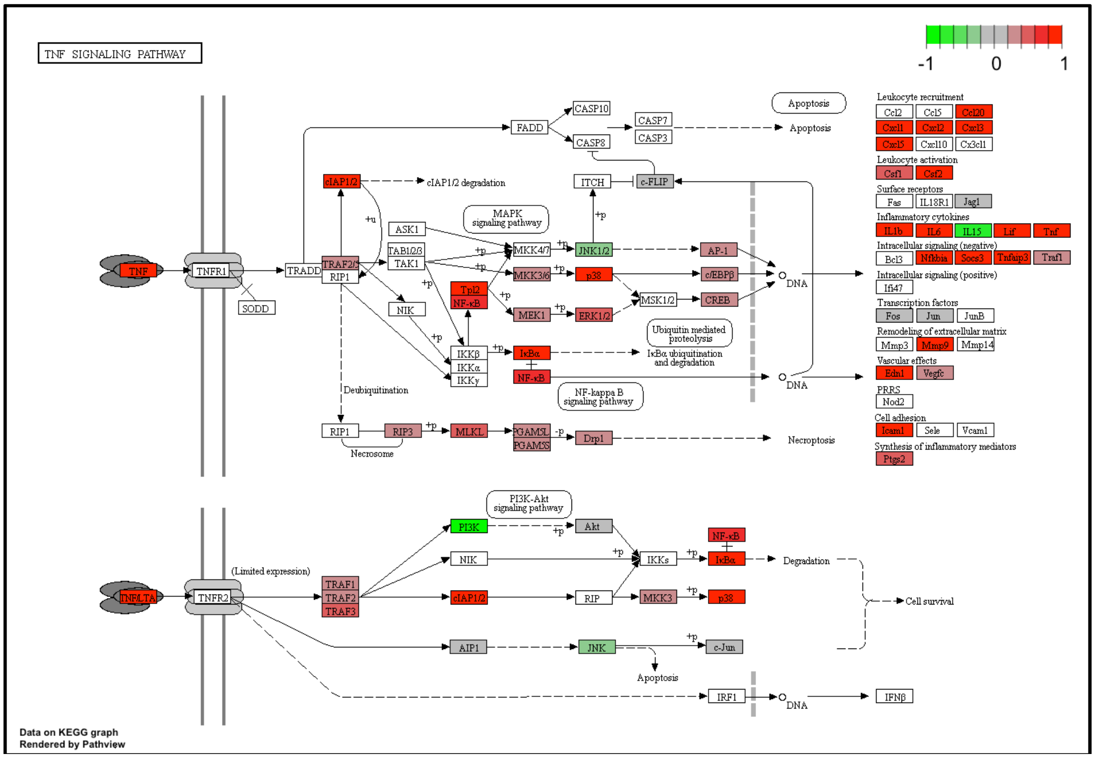

TNF SIGNALING PATHWAY
-1
0
1
TNF
TNFR1
SODD
TRADD
TRAF2/5
RIP1
cIAP1/2
cIAP1/2 degradation
FADD
CASP10
CASP8
CASP7
CASP3
Apoptosis
ITCH
c-FLIP
ASK1
TAB1/2/3
TAK1
MAPK signaling pathway
MKK4/7
MKK3/6
JNK1/2
p38
AP-1
c/EBPβ
MSK1/2
CREB
Tpl2
NF-κB
MEK1
ERK1/2
NIK
IKKβ
IKKα
IKKγ
IκBα
Ubiquitin mediated proteolysis
IκBα ubiquitination and degradation
NF-kappa B signaling pathway
DNA
Deubiquitination
RIP3
MLKL
PGAM5L
PGAM5S
Drp1
Necroptosis
Necrosome
Leukocyte recruitment
Ccl2
Ccl5
Ccl20
Cxcl1
Cxcl2
Cxcl3
Cxcl5
Cxcl10
Cx3cl1
Leukocyte activation
Csf1
Csf2
Surface receptors
Fas
IL18R1
Jag1
Inflammatory cytokines
IL1b
IL6
IL15
Lif
Tnf
Intracellular signaling (negative)
Bcl3
Nfkbia
Socs3
Tnfaip3
Traf1
Intracellular signaling (positive)
Ifi47
Transcription factors
Fos
Jun
JunB
Remodeling of extracellular matrix
Mmp3
Mmp9
Mmp14
Vascular effects
Edn1
Vegfc
PRRS
Nod2
Cell adhesion
Icam1
Sele
Vcam1
Synthesis of inflammatory mediators
Ptgs2
PI3K-Akt signaling pathway
PI3K
Akt
TNF/LTA
TNFR2
(Limited expression)
TRAF1
TRAF2
TRAF3
IKKs
MKK3
RIP
Degradation
Cell survival
AIP1
JNK
c-Jun
IRF1
IFNβ
Data on KEGG graph
Rendered by Pathview

(c)

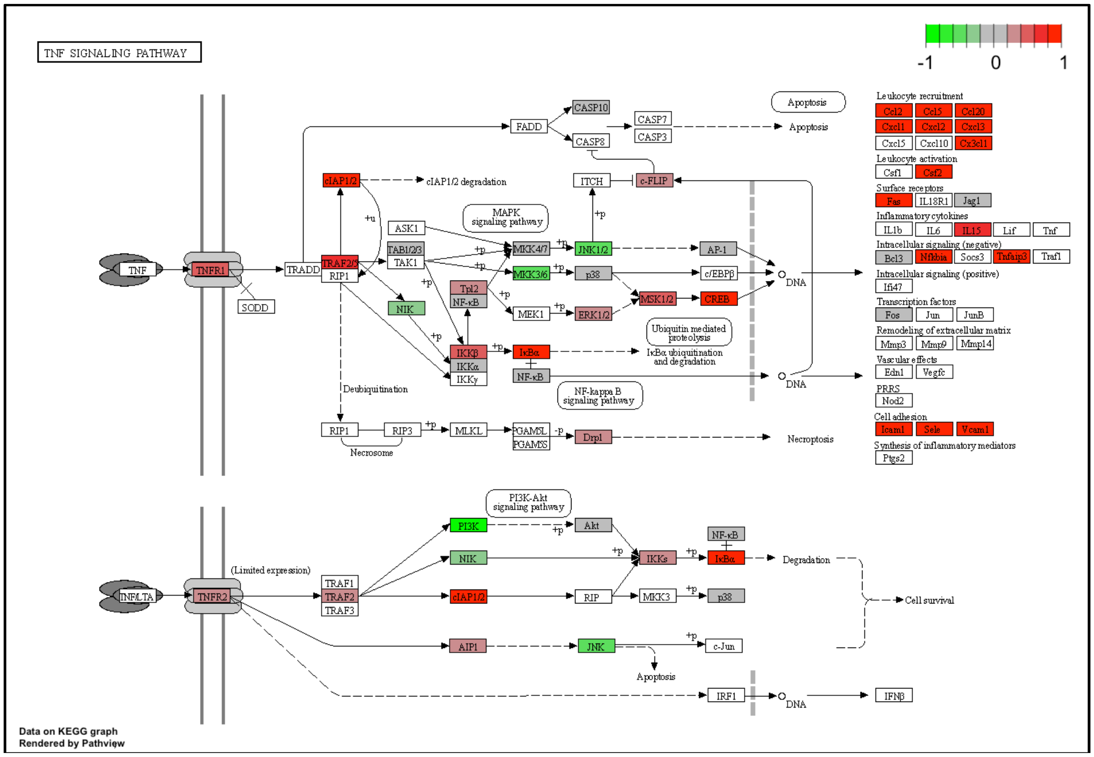

TNF SIGNALING PATHWAY
-1
0
1
TNF
TNFR1
SODD
TRADD
TRAF2/5
RIP1
cIAP1/2
cIAP1/2 degradation
FADD
CASP10
CASP8
CASP7
CASP3
Apoptosis
ITCH
c-FLIP
ASK1
TAB1/2/3
TAK1
MAPK signaling pathway
MKK4/7
MKK3/6
JNK1/2
p38
AP-1
c/EBPβ
MSK1/2
CREB
Tpl2
NF-κB
MEK1
ERK1/2
NIK
IKKβ
IKKα
IKKγ
IκBα
Ubiquitin mediated proteolysis
IκBα ubiquitination and degradation
NF-kappa B signaling pathway
DNA
Deubiquitination
RIP3
MLKL
PGAM5L
PGAM5S
Drp1
Necroptosis
Necrosome
Leukocyte recruitment
Ccl2
Ccl5
Ccl20
Cxcl1
Cxcl2
Cxcl3
Cxcl5
Cxcl10
Cx3cl1
Leukocyte activation
Csf1
Csf2
Surface receptors
Fas
IL18R1
Jag1
Inflammatory cytokines
IL1b
IL6
IL15
Lif
Tnf
Intracellular signaling (negative)
Bcl3
Nfkbia
Socs3
Tnfaip3
Traf1
Intracellular signaling (positive)
Ifi47
Transcription factors
Fos
Jun
JunB
Remodeling of extracellular matrix
Mmp3
Mmp9
Mmp14
Vascular effects
Edn1
Vegfc
PRRS
Nod2
Cell adhesion
Icam1
Sele
Vcam1
Synthesis of inflammatory mediators
Ptgs2
PI3K-Akt signaling pathway
PI3K
Akt
TNF/LTA
TNFR2
(Limited expression)
TRAF1
TRAF2
TRAF3
IKKs
MKK3
RIP
Degradation
Cell survival
AIP1
JNK
c-Jun
IRF1
IFNβ
Data on KEGG graph
Rendered by Pathview

Figure S11. DEGs in HIEC cells infected with PDCoV **(a),** NHBE cells infected with SARS-CoV-2 **(b)** and IPEC cells infected with SADS-CoV **(c)** at 24 hpi in **Malaria signaling pathway.**

(a)

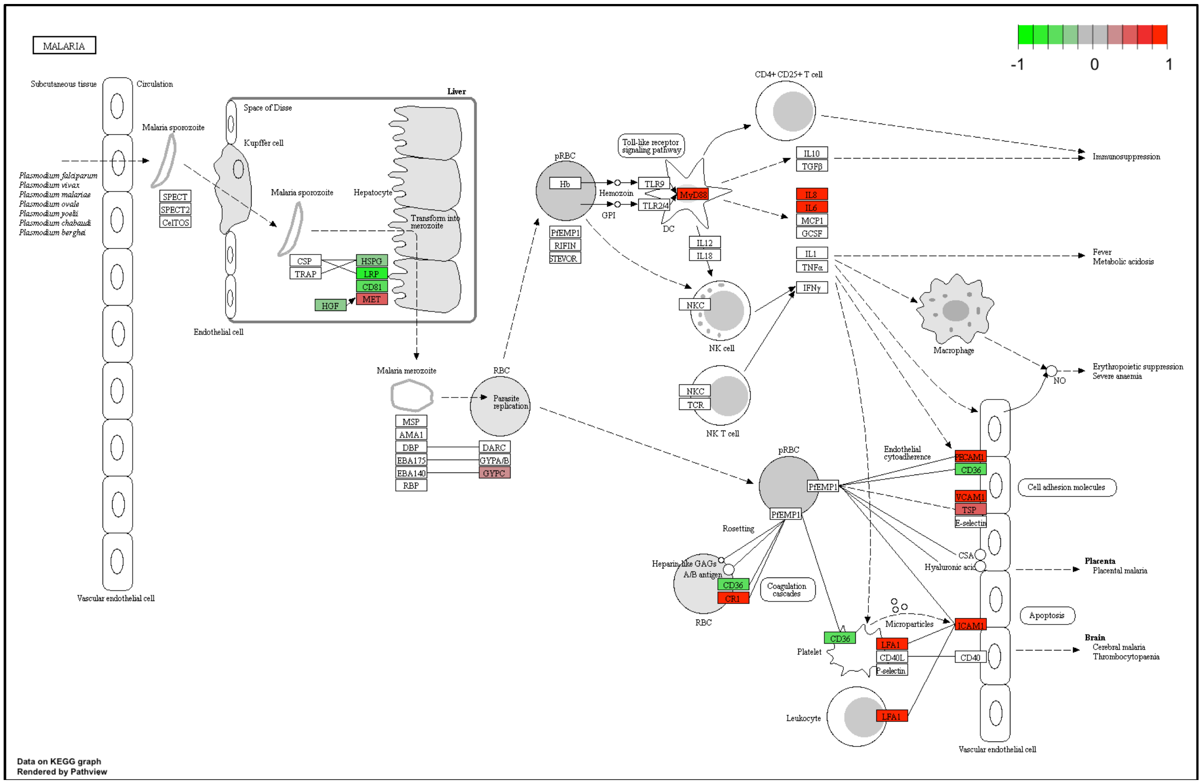

(b)

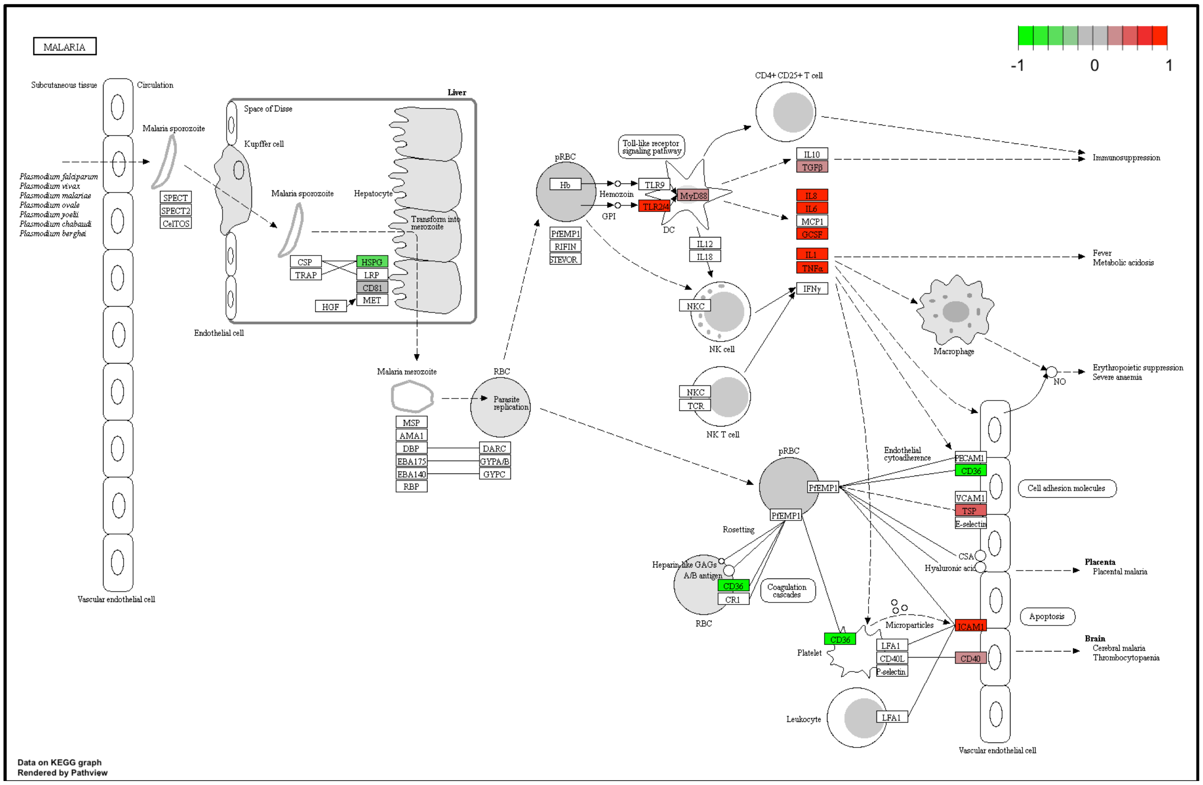
MALARIA
-1
0
1
Subcutaneous tissue
Circulation
Malaria sporozoite
Plasmodium falciparum
Plasmodium vivax
Plasmodium malariae
Plasmodium ovale
Plasmodium yoelii
Plasmodium chabaudi
Plasmodium berghei
SPECT
SPECT2
CelTOS
Liver
Space of Disse
Kupffer cell
Malaria sporozoite
Hepatocyte
Transform into merozoite
CSP
TRAP
HSPG
LRP
CD81
MET
HGF
Endothelial cell
Vascular endothelial cell
Malaria merozoite
MSP
AMA1
DBP
EBA175
EBA140
RBP
DARC
GYPA/B
GYPC
RBC
Parasite replication
pRBC
Hb
Hemozoin
GPI
PfEMP1
RIFIN
STEVOR
Toll-like receptor signaling pathway
TLR9
TLR2/4
MyD88
DC
IL12
IL18
CD4+ CD25+ T cell
IL10
TGFβ
Immunosuppression
IL8
IL6
MCP1
GCSF
IL1
TNFα
IFNγ
Fever
Metabolic acidosis
NKC
NK cell
NKC
TCR
NK T cell
Macrophage
NO
Erythropoietic suppression
Severe anaemia
pRBC
PfEMP1
PfEMP1
Endothelial cytoadherence
PECAM1
CD36
VCAM1
TSP
E-selectin
Cell adhesion molecules
Rosetting
Heparin-like GAGs
A/B antigen
CD36
CR1
Coagulation cascades
RBC
CSA
Hyaluronic acid
Placenta
Placental malaria
Apoptosis
Microparticles
ICAM1
CD36
Platelet
LFA1
CD40L
P-selectin
CD40
Brain
Cerebral malaria
Thrombocytopaenia
Leukocyte
LFA1
Vascular endothelial cell
Data on KEGG graph
Rendered by Pathview

(c)

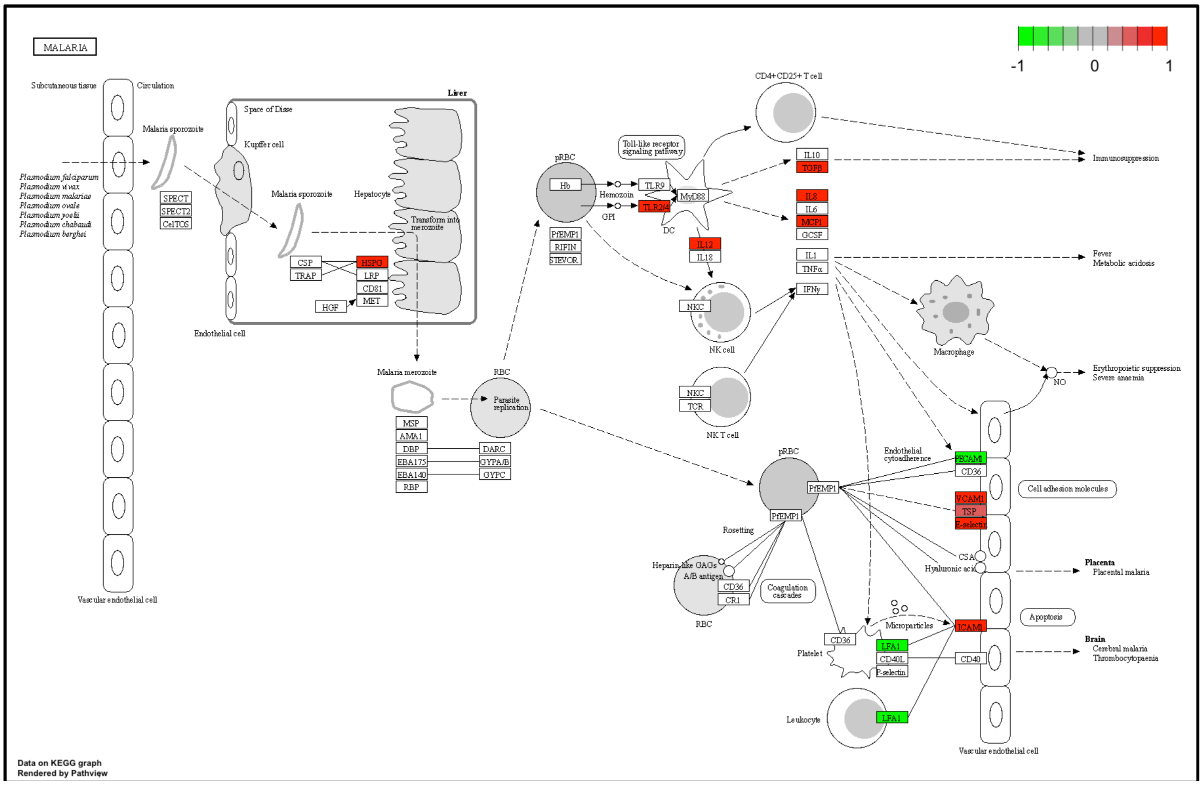
MALARIA
-1
0
1
Subcutaneous tissue
Circulation
Malaria sporozoite
Plasmodium falciparum
Plasmodium vivax
Plasmodium malariae
Plasmodium ovale
Plasmodium yoelii
Plasmodium chabaudi
Plasmodium berghei
SPECT
SPECT2
CelTOS
Liver
Space of Disse
Kupffer cell
Malaria sporozoite
Hepatocyte
Transform into merozoite
CSP
TRAP
HSPG
LRP
CD81
MET
HGF
Endothelial cell
Vascular endothelial cell
Malaria merozoite
MSP
AMA1
DBP
EBA175
EBA140
RBP
DARC
GYPA/B
GYPC
RBC
Parasite replication
pRBC
Hb
Hemozoin
GPI
PfEMP1
RIFIN
STEVOR
Toll-like receptor signaling pathway
TLR9
TLR2/4
MyD88
DC
IL12
IL18
CD4+ CD25+ T cell
IL10
TGFβ
Immunosuppression
IL8
IL6
MCP1
GCSF
IL1
TNFα
IFNγ
Fever
Metabolic acidosis
NKC
NK cell
NKC
TCR
NK T cell
Macrophage
NO
Erythropoietic suppression
Severe anaemia
pRBC
PfEMP1
PfEMP1
Endothelial cytoadherence
PECAM1
CD36
VCAM1
TSP
E-selectin
Cell adhesion molecules
Rosetting
Heparin-like GAGs
A/B antigen
CD36
CR1
Coagulation cascades
RBC
CSA
Hyaluronic acid
Placenta
Placental malaria
Apoptosis
Microparticles
ICAM1
CD36
Platelet
LFA1
CD40L
P-selectin
CD40
Brain
Cerebral malaria
Thrombocytopaenia
Leukocyte
LFA1
Vascular endothelial cell
Data on KEGG graph
Rendered by Pathview

Figure S12. DEGs in HIEC cells infected with PDCoV **(a),** NHBE cells infected with SARS-CoV-2 **(b)** and IPEC cells infected with SADS-CoV **(c)** at 24 hpi in **Influenza A.**

(a)

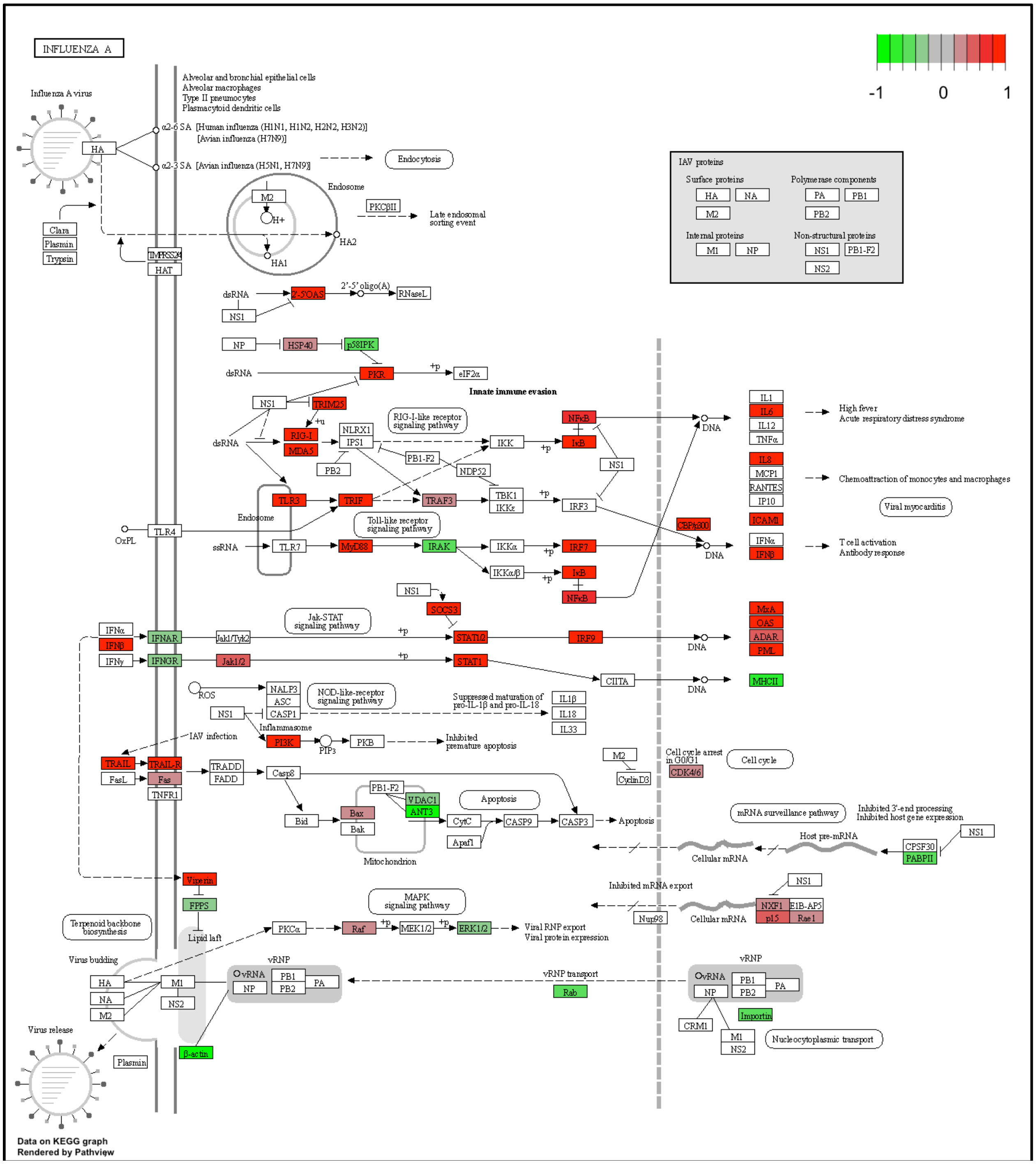

(b)

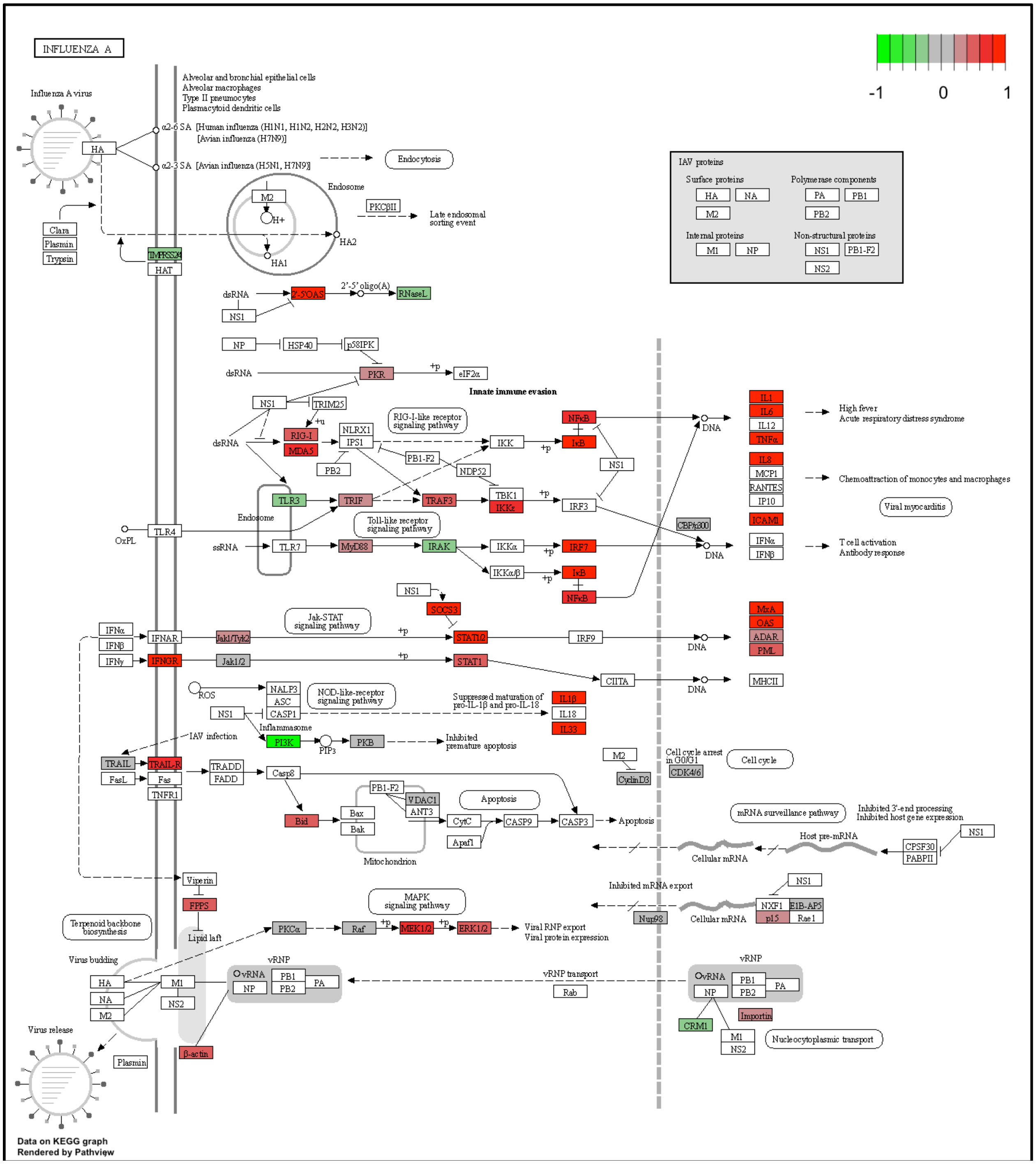
INFLUENZA A
-1
0
1
Influenza A virus
Alveolar and bronchial epithelial cells
Alveolar macrophages
Type II pneumocytes
Plasmacytoid dendritic cells
HA
α2-6 SA [Human influenza (H1N1, H1N2, H2N2, H3N2)]
[Avian influenza (H7N9)]
α2-3 SA [Avian influenza (H5N1, H7N9)]
Endocytosis
Endosome
M2
H+
HA2
HA1
PKCβII
Late endosomal sorting event
Clara
Plasmin
Trypsin
TMPRSS2/4
HAT
IAV proteins
Surface proteins
HA
NA
M2
Polymerase components
PA
PB1
PB2
Internal proteins
M1
NP
Non-structural proteins
NS1
PB1-F2
NS2
dsRNA
2'-5'OAS
2'-5' oligo(A)
RNaseL
NS1
NP
HSP40
p58IPK
PKR
+p
eIF2α
Innate immune evasion
TRIM25
+u
RIG-I
MDA5
NLRX1
IPS1
PB1-F2
PB2
RIG-I-like receptor signaling pathway
IKK
NFκB
IκB
NS1
NDP52
TBK1
IKKε
IRF3
TRAF3
TRIF
TLR3
Endosome
Toll-like receptor signaling pathway
TLR4
OxPL
ssRNA
TLR7
MyD88
IRAK
IKKα
IRF7
IKKα/β
IκB
NFκB
CBP/p300
DNA
IL1
IL6
IL12
TNFα
High fever
Acute respiratory distress syndrome
IL8
MCP1
RANTES
IP10
Chemoattraction of monocytes and macrophages
Viral myocarditis
ICAM1
IFNα
IFNβ
T cell activation
Antibody response
NS1
SOCS3
Jak-STAT signaling pathway
IFNα
IFNβ
IFNγ
IFNAR
IFNGR
Jak1/Tyk2
Jak1/2
STAT1/2
STAT1
IRF9
MxA
OAS
ADAR
PML
CIITA
MHCII
ROS
NALP3
ASC
CASP1
NS1
Inflammasome
NOD-like-receptor signaling pathway
Suppressed maturation of pro-IL-1β and pro-IL-18
IL1β
IL18
IL33
IAV infection
PI3K
PIP3
PKB
Inhibited premature apoptosis
M2
CyclinD3
Cell cycle arrest in G0/G1
CDK4/6
Cell cycle
TRAIL
TRAIL-R
FasL
Fas
TNFR1
TRADD
FADD
Casp8
PB1-F2
VDAC1
ANT3
Apoptosis
Bid
Bax
Bak
CytC
Apaf1
CASP9
CASP3
Apoptosis
Mitochondrion
mRNA surveillance pathway
Inhibited 3'-end processing
Inhibited host gene expression
NS1
Host pre-mRNA
CPSF30
PABPII
Cellular mRNA
Inhibited mRNA export
NS1
NXF1
E1B-AP5
p15
Rae1
Nup98
Cellular mRNA
Viperin
FPPS
Lipid raft
MAPK signaling pathway
Terpenoid backbone biosynthesis
PKCα
Raf
MEK1/2
ERK1/2
Viral RNP export
Viral protein expression
Virus budding
vRNP
vRNA
NP
PB1
PB2
PA
HA
NA
M2
M1
NS2
vRNP transport
Rab
vRNP
vRNA
NP
PB1
PB2
PA
CRM1
Importin
M1
NS2
Nucleocytoplasmic transport
Virus release
Plasmin
β-actin
Data on KEGG graph
Rendered by Pathview

(c)

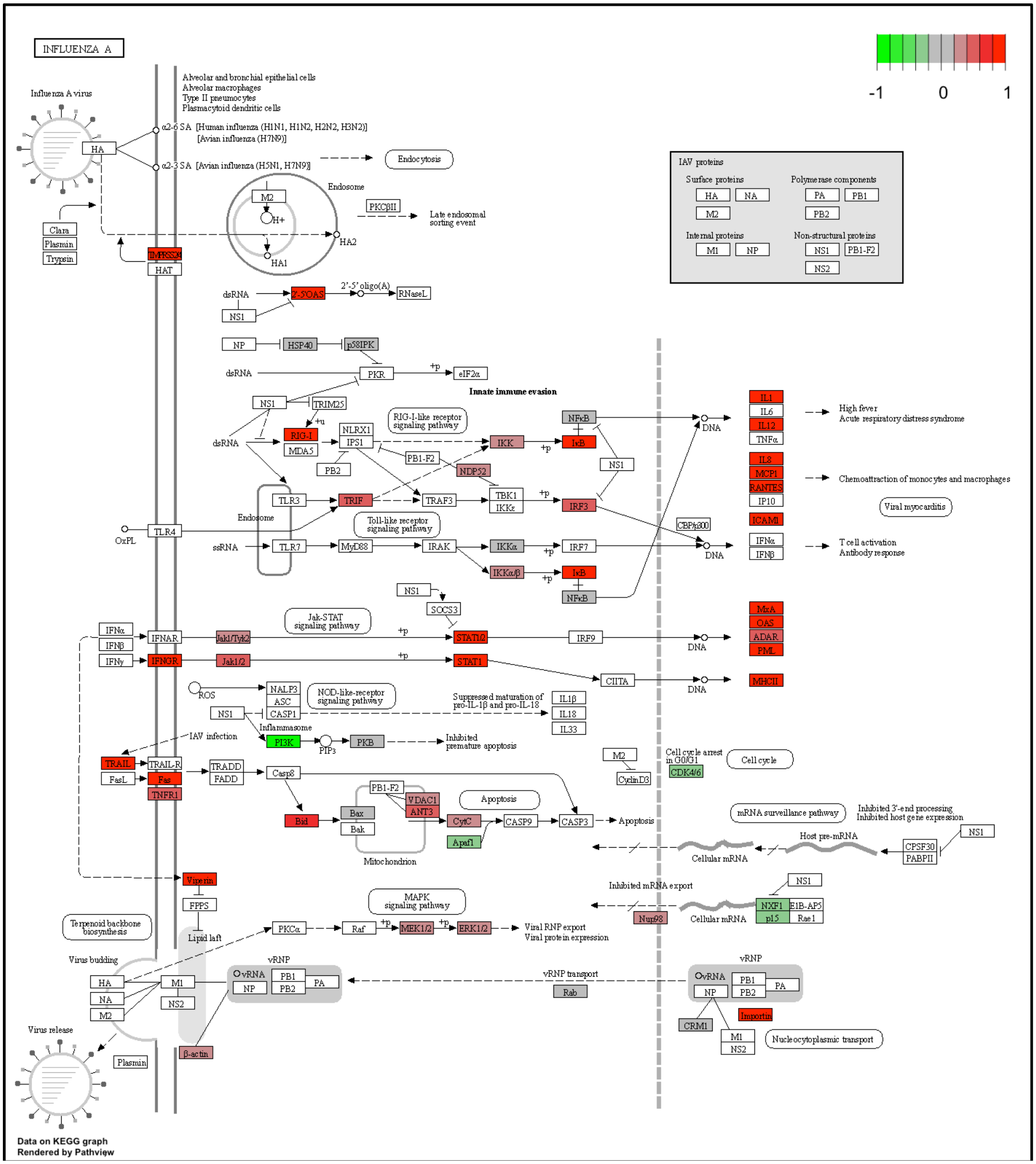
INFLUENZA A
-1
0
1
Influenza A virus
Alveolar and bronchial epithelial cells
Alveolar macrophages
Type II pneumocytes
Plasmacytoid dendritic cells
α2-6 SA [Human influenza (H1N1, H1N2, H2N2, H3N2)]
[Avian influenza (H7N9)]
α2-3 SA [Avian influenza (H5N1, H7N9)]
Endocytosis
Endosome
Late endosomal sorting event
IAV proteins
Surface proteins
Polymerase components
Internal proteins
Non-structural proteins
Innate immune evasion
High fever
Acute respiratory distress syndrome
Chemoattraction of monocytes and macrophages
Viral myocarditis
T cell activation
Antibody response
RIG-I-like receptor signaling pathway
Toll-like receptor signaling pathway
Jak-STAT signaling pathway
NOD-like-receptor signaling pathway
Suppressed maturation of pro-IL-1β and pro-IL-18
Inflammasome
IAV infection
Inhibited premature apoptosis
Cell cycle arrest in G0/G1
Cell cycle
Apoptosis
Mitochondrion
mRNA surveillance pathway
Inhibited 3'-end processing
Inhibited host gene expression
Host pre-mRNA
Cellular mRNA
Inhibited mRNA export
MAPK signaling pathway
Viral RNP export
Viral protein expression
Terpenoid backbone biosynthesis
Lipid raft
Virus budding
Virus release
vRNP
vRNP transport
Nucleocytoplasmic transport
Data on KEGG graph
Rendered by Pathvisio

Figure S13. DEGs in HIEC cells infected with PDCoV **(a),** NHBE cells infected with SARS-CoV-2 **(b)** and IPEC cells infected with SADS-CoV **(c)** at 24 hpi in **Coronavirus Disease.**

(a)

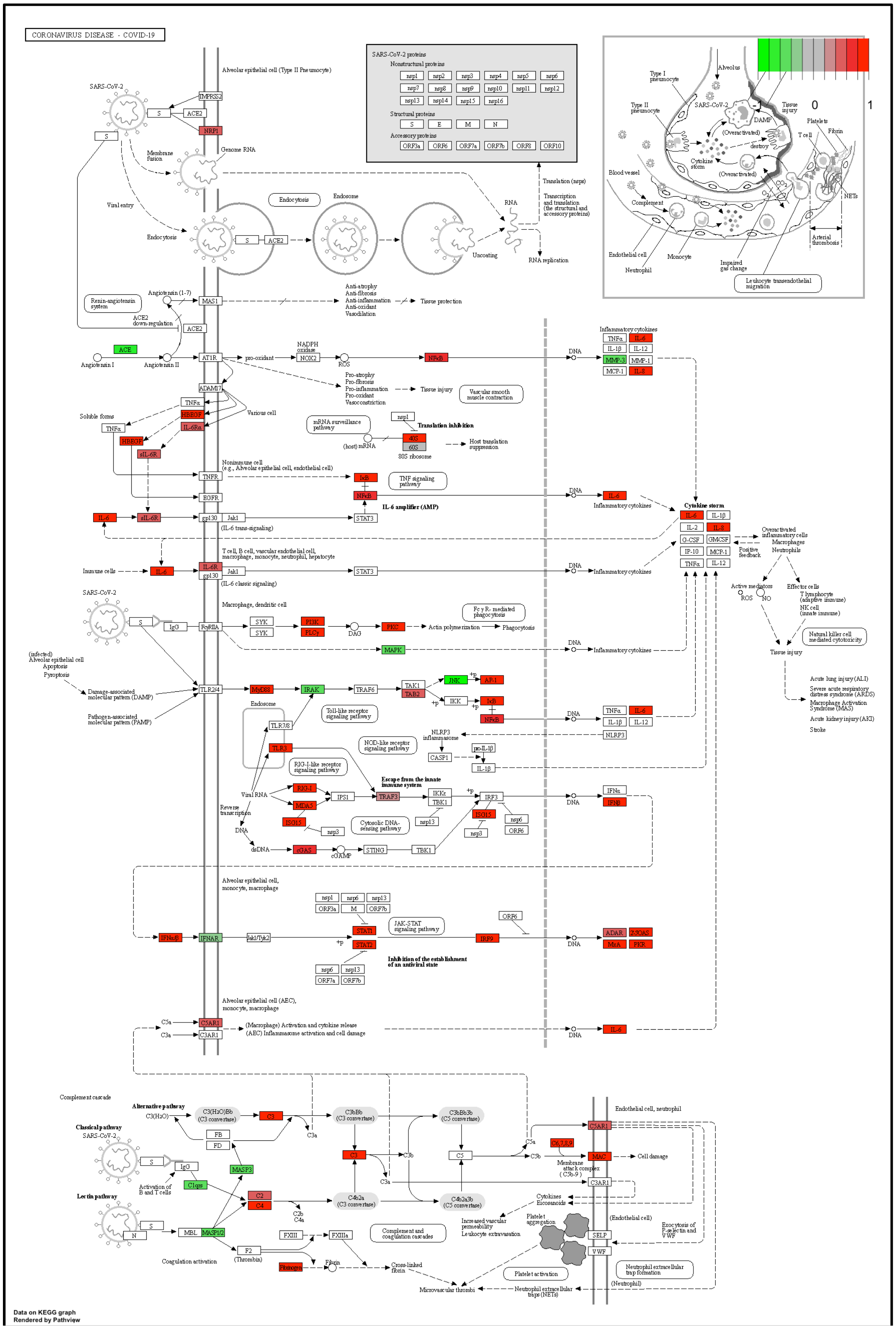

(b)

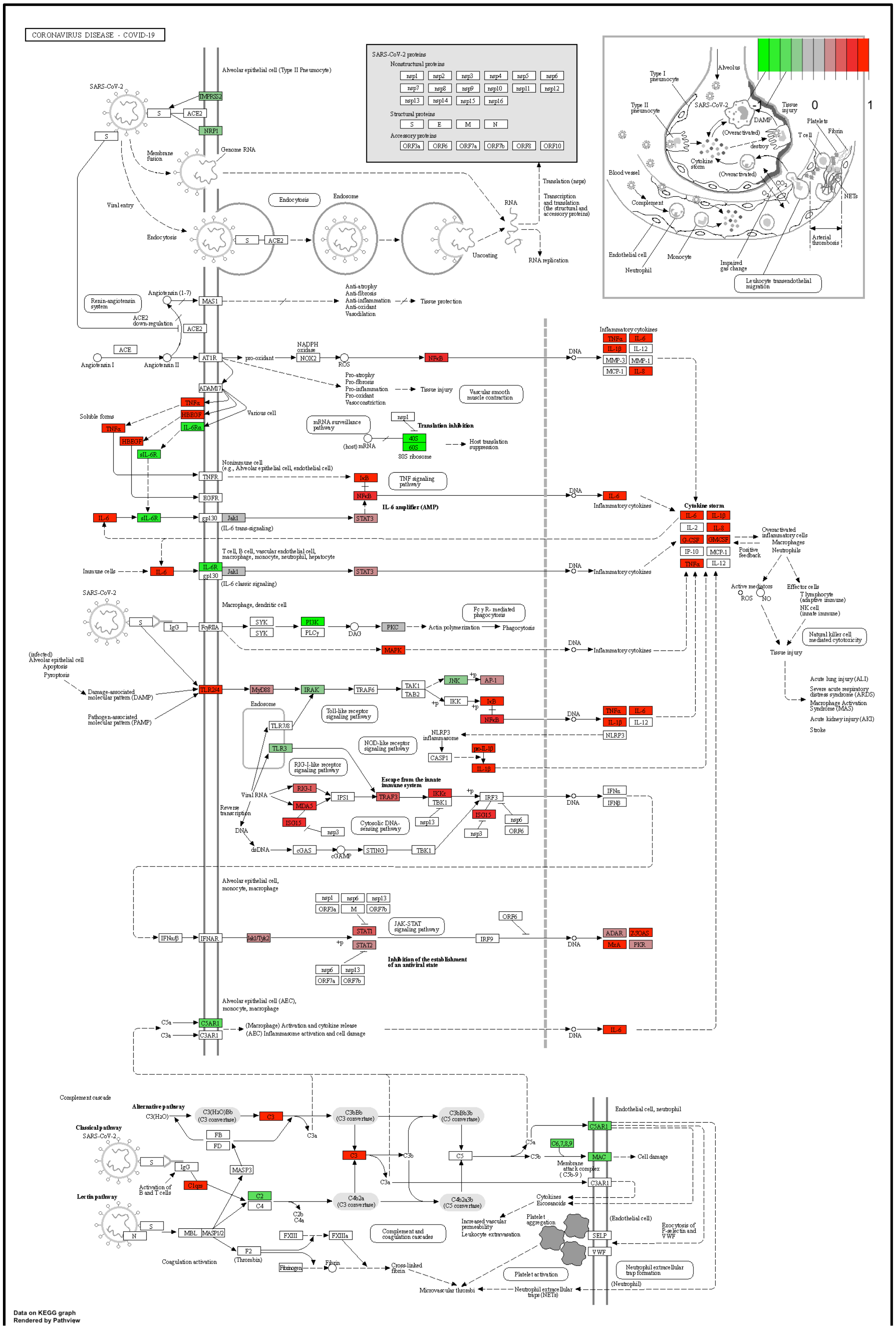
CORONAVIRUS DISEASE - COVID-19
SARS-CoV-2 proteins
Nonstructural proteins
Structural proteins
Accessory proteins
Alveolar epithelial cell (Type II Pneumocyte)
Endocytosis
Endosome
Uncoating
RNA replication
Tissue protection
Inflammatory cytokines
Cytokine storm
IL-6 amplifier (AMP)
Translation inhibition
Escape from the innate immune system
Inhibition of the establishment of an antiviral state
Complement cascade
Alternative pathway
Classical pathway
Lectin pathway
Coagulation activation
Microvascular thrombi
Data on KEGG graph
Rendered by Pathview

(c)

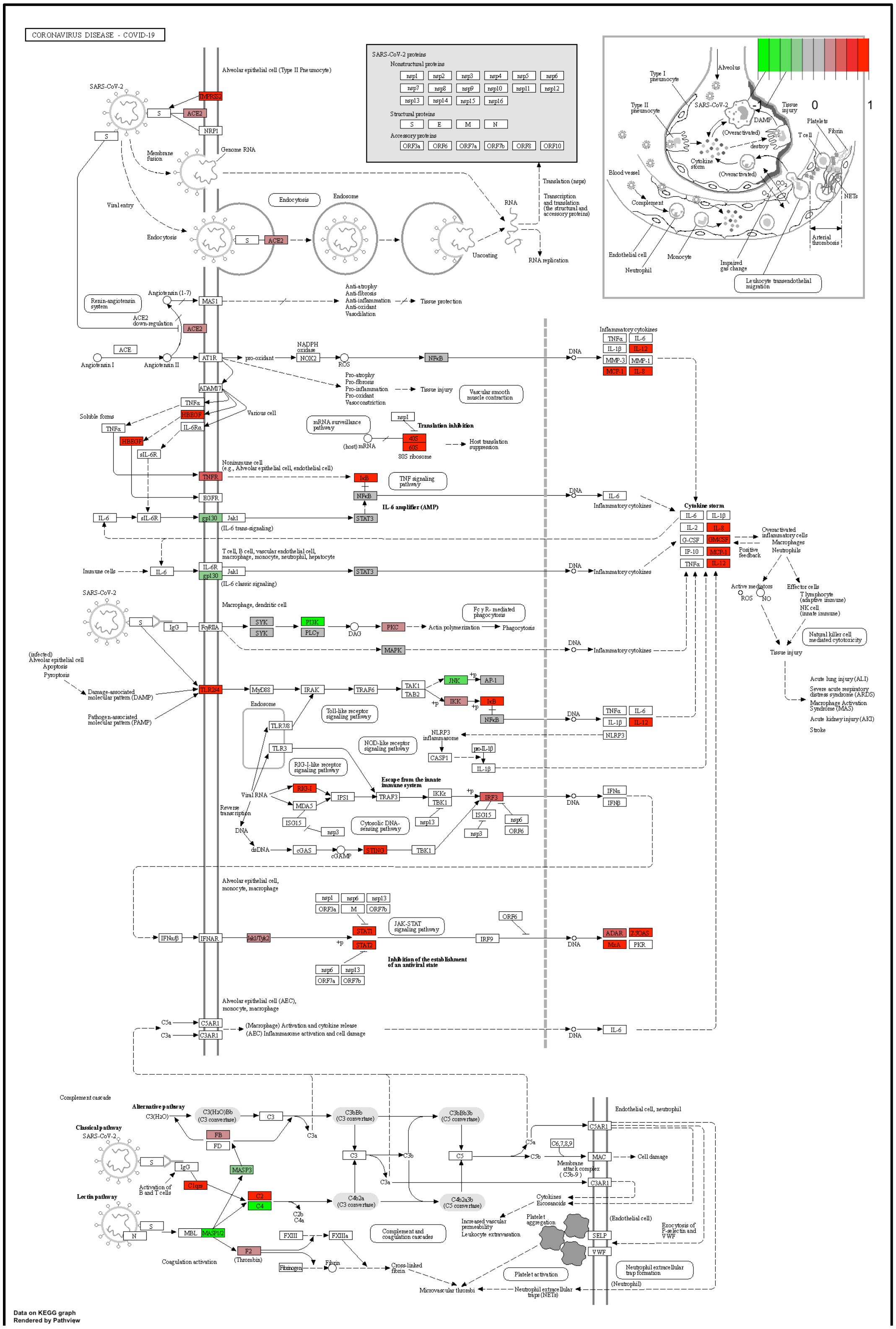

Figure S14. Common DE genes that are shared among PDCoV, SARS-CoV-2 and SADS-CoV infections in upregulated (up) and downregulated (down) genes.

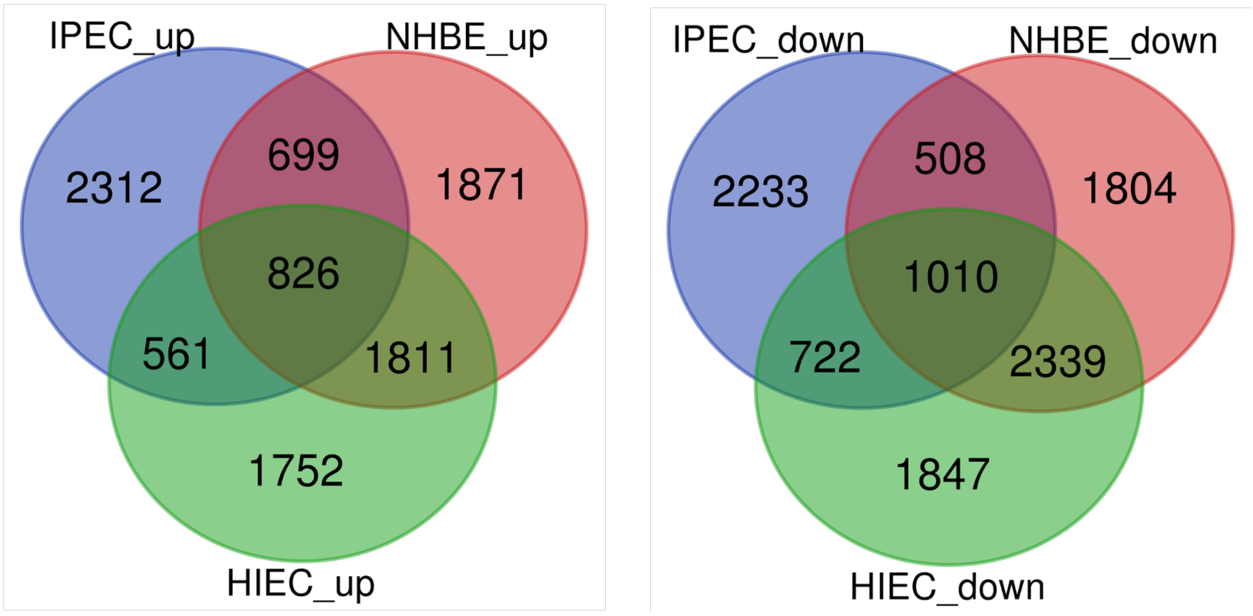

Figure S15. Common DE genes that are shared among PDCoV, SARS-CoV-2 and SADS-CoV infections at 24 hpi across 10 pathways in upregulated genes*.

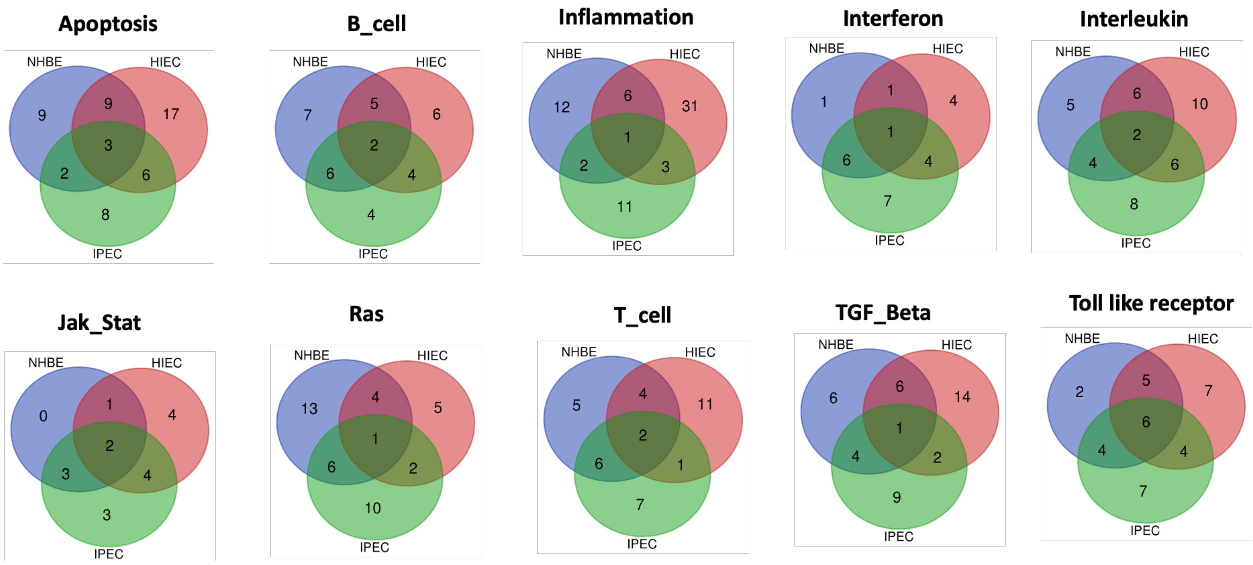

*Apoptosis signaling pathway, B cell activation, Inflammation/cytokine signaling pathway, Interferon signaling pathway, Interleukin signaling pathway, Jak-Stat signaling pathway, Ras signaling pathway, T cell activation, TGF-β signaling pathway, Toll like receptor signaling pathway and Ras signaling pathway.

Figure S16. Common DE genes that are shared among PDCoV, SARS-CoV-2 and SADS-CoV infections at 24 hpi across 10 pathways in downregulated genes*.

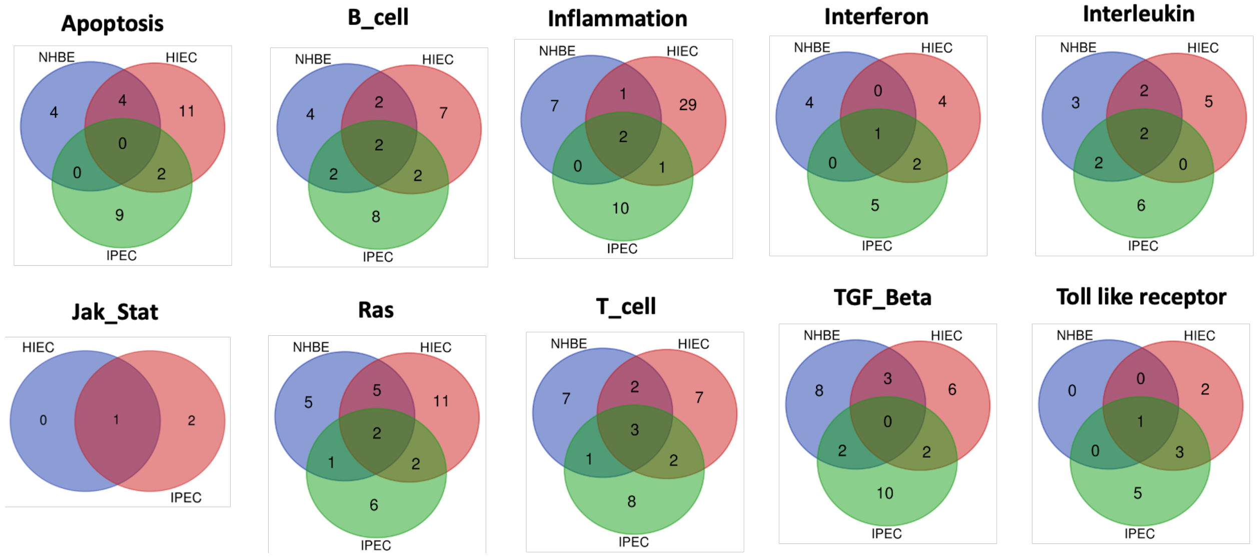

*Apoptosis signaling pathway, B cell activation, Inflammation/cytokine signaling pathway, Interferon signaling pathway, Interleukin signaling pathway, Jak-Stat signaling pathway, Ras signaling pathway, T cell activation, TGF-β signaling pathway, Toll like receptor signaling pathway and Ras signaling pathway.

Figure S17. Common DE genes that are upregulated in HIEC cells infected with PDCoV and downregulated in NHBE cells infected with SARS-CoV-2 at 24 hpi across 9 pathways.

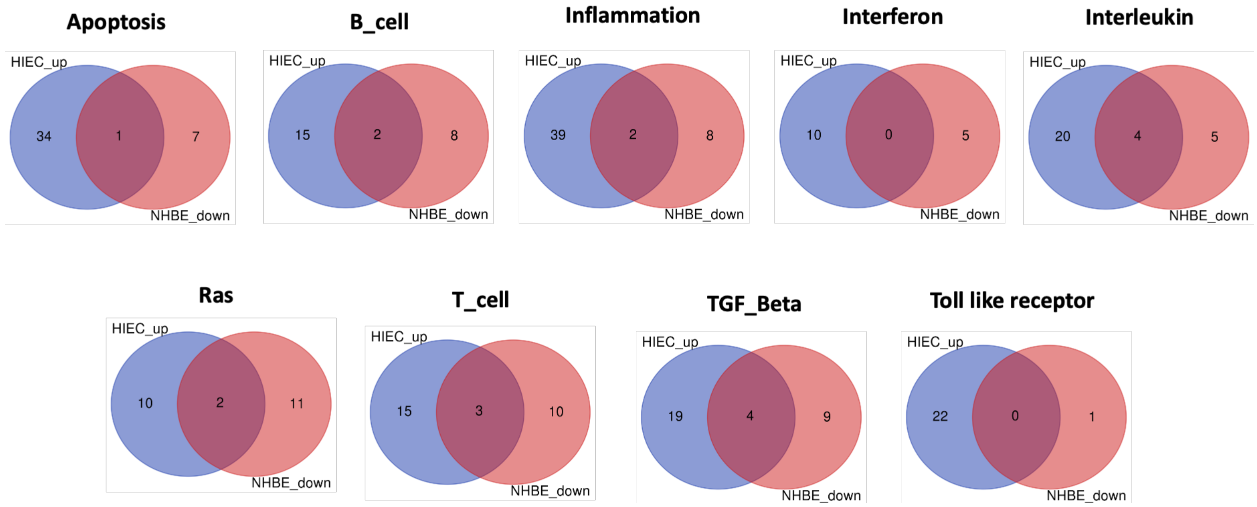

Figure S18. Common DE genes that are upregulated in NHBE cells infected with PDCoV and downregulated in HIEC cells infected with SARS-CoV-2 at 24 hpi across 10 pathways.

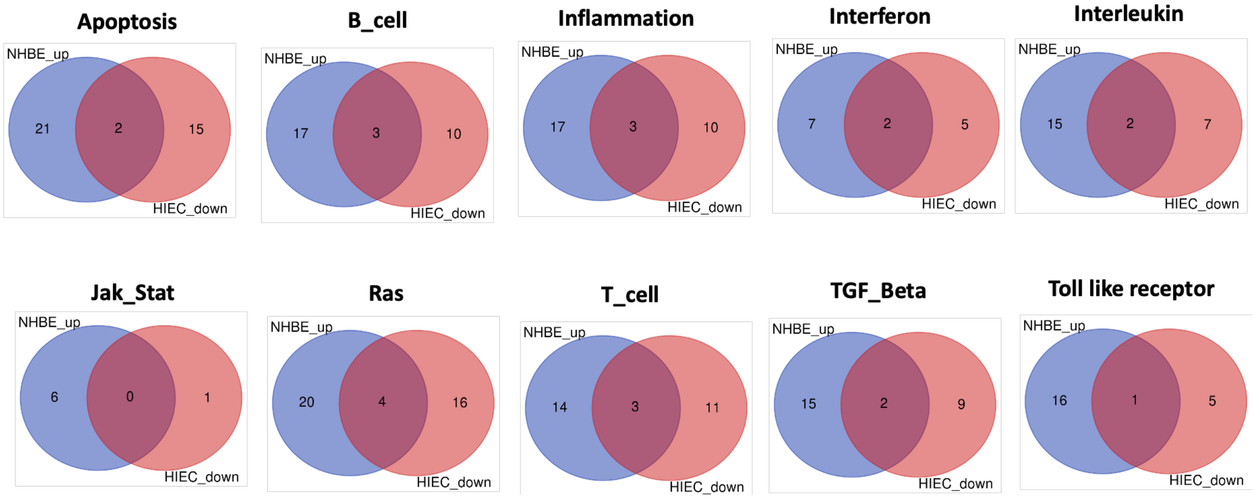

Figure S19. Correlation of HIEC cells infected with PDCoV and NHBE cell infected with SARS-CoV-2.

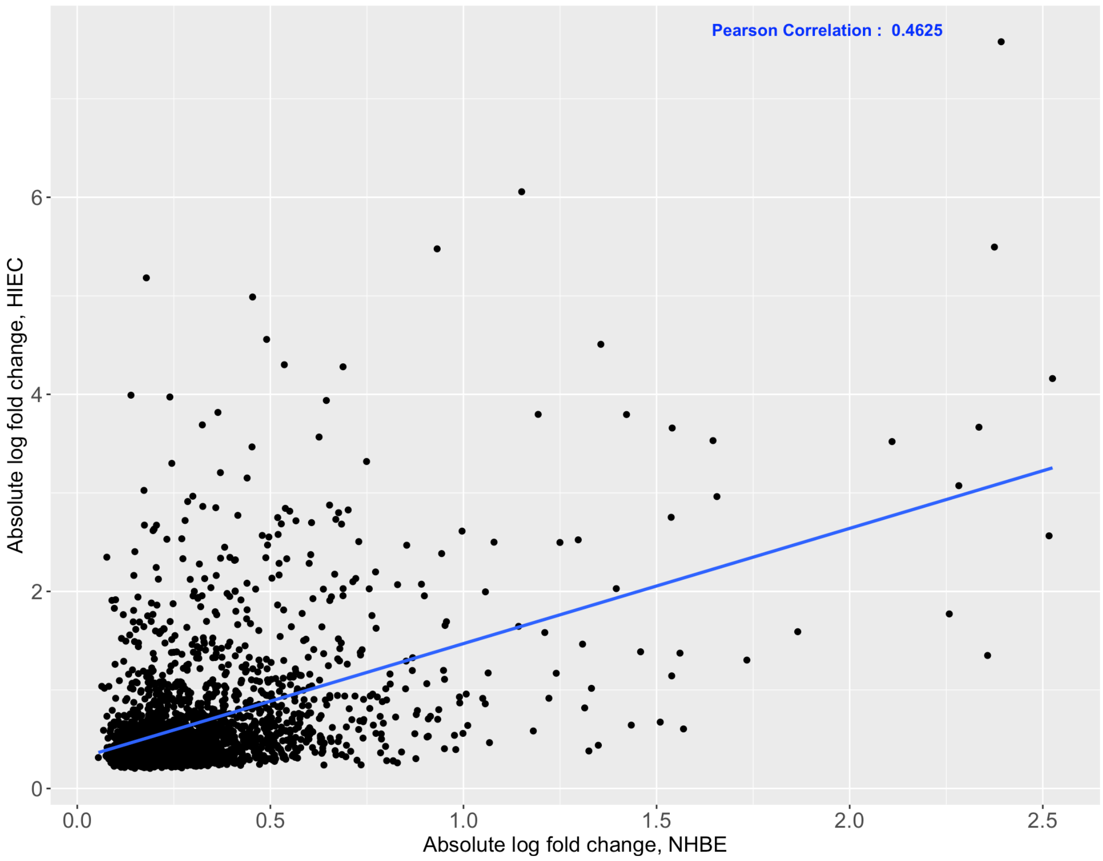

Table S1. Description of common DE genes that are shared among PDCoV, SARS-CoV-2 and SADS-CoV infections at 24 hpi across 10 pathways in **(a)** Upregulated genes according to Venn diagrams in **Figure S15** and **(b)** downregulated genes according to Venn diagrams in **Figure S16. (c)** Upregulated DE genes in HIEC cells and downregulated genes in NHBE cells according to Venn diagrams in **Figure S17**. **(d)** Downregulated DE genes in HIEC cells and Upregulated genes in NHBE cells according to Venn diagrams in **Figure S18**. **(e)** Upregulated DE genes in HIEC cells and downregulated genes in NHBE cells. **(f)** Downregulated DE genes in HIEC cells and Upregulated genes in NHBE cells.
